# Acod1/itaconate activates Nrf2 in pulmonary microvascular endothelial cells to protect against the obesity-induced pulmonary microvascular endotheliopathy

**DOI:** 10.1186/s12931-024-02827-w

**Published:** 2024-05-10

**Authors:** Li Zhu, Zhuhua Wu, Yingli Liu, Yue Ming, Pei Xie, Miao Jiang, Yong Qi

**Affiliations:** 1 Department of Pulmonary and Critical Care Medicine, Zhengzhou University People’s Hospital, Henan Provincial People’s Hospital, Zhengzhou, Henan People’s Republic of China; 2 Academy of Medical Science, Zhengzhou University, Zhengzhou, Henan People’s Republic of China; 3 Department of Pulmonary and Critical Care Medicine, Henan University People’s Hospital, Henan Provincial People’s Hospital, Zhengzhou, Henan People’s Republic of China; 4 Department of Pulmonary and Critical Care Medicine, Henan Provincial People’s Hospital, Zhengzhou University People’s Hospital, Henan University People’s Hospital, Zhengzhou, Henan People’s Republic of China

**Keywords:** Acod1, 4-OI, Obesity, PMVECs, Nrf2

## Abstract

**Background:**

Obesity is the main risk factor leading to the development of various respiratory diseases, such as asthma and pulmonary hypertension. Pulmonary microvascular endothelial cells (PMVECs) play a significant role in the development of lung diseases. Aconitate decarboxylase 1 (Acod1) mediates the production of itaconate, and Acod1/itaconate axis has been reported to play a protective role in multiple diseases. However, the roles of Acod1/itaconate axis in the PMVECs of obese mice are still unclear.

**Methods:**

mRNA-seq was performed to identify the differentially expressed genes (DEGs) between high-fat diet (HFD)-induced PMVECs and chow-fed PMVECs in mice (|log_2_ fold change| ≥ 1, *p* ≤ 0.05). Free fatty acid (FFA) was used to induce cell injury, inflammation and mitochondrial oxidative stress in mouse PMVECs after transfection with the Acod1 overexpressed plasmid or 4-Octyl Itaconate (4-OI) administration. In addition, we investigated whether the nuclear factor erythroid 2-like 2 (Nrf2) pathway was involved in the effects of Acod1/itaconate in FFA-induced PMVECs.

**Results:**

Down-regulated Acod1 was identified in HFD mouse PMVECs by mRNA-seq. Acod1 expression was also reduced in FFA-treated PMVECs. Acod1 overexpression inhibited cell injury, inflammation and mitochondrial oxidative stress induced by FFA in mouse PMVECs. 4-OI administration showed the consistent results in FFA-treated mouse PMVECs. Moreover, silencing Nrf2 reversed the effects of Acod1 overexpression and 4-OI administration in FFA-treated PMVECs, indicating that Nrf2 activation was required for the protective effects of Acod1/itaconate.

**Conclusion:**

Our results demonstrated that Acod1/Itaconate axis might protect mouse PMVECs from FFA-induced injury, inflammation and mitochondrial oxidative stress via activating Nrf2 pathway. It was meaningful for the treatment of obesity-caused pulmonary microvascular endotheliopathy.

**Supplementary Information:**

The online version contains supplementary material available at 10.1186/s12931-024-02827-w.

## Introduction

Obesity, a global health issue, is becoming increasingly prevalent due to sedentary lifestyles, over-nutrition and rapidly aging population [1]. Obesity is the main risk factor for the development of numerous diseases, such as type 2 diabetes, cardiovascular diseases, cardiomyopathy and lung diseases, which have seriously threatened human health [2–5]. Particularly, it has been reported that obesity influences lung homeostasis via mass loading, hormonal, metabolic and inflammatory factors [5]. Pulmonary microvascular endothelial cells (PMVECs), lining in the blood vessel wall, maintain internal homeostasis and physiological and immunological functions as well as mediate the development of lung diseases [6, 7]. Multiple studies have shown that obesity leads to microvascular endothelial dysfunction [8]. Herein, PMVECs were applied to explore the molecular mechanism by which obesity affected pulmonary microvascular endotheliopathy.

It is widely known that tricarboxylic acid (TCA) cycle exerts crucial roles in immunity and inflammation [9–11]. Itaconate, a product of decarboxylation of cis-aconitate mediated by Aconitate decarboxylase 1 (Acod1) in TCA cycle, attracts much attention due to the capacity to control inflammation and induce immune tolerance [12–14]. *Acod1*, also known as *immune-responsive gene 1* (*Irg1*), is first identified as a 2.3 kilobase (kb) cDNA from mouse macrophages induced by lipopolysaccharides (LPS) [15]. It is emerging as a regulator of immunometabolism in inflammation and infection [16]. The anti-inflammatory roles of Acod1 and Itaconate have been reported widely. For instance, it has been observed that Acod1 deficiency makes mouse weight lose rapidly and enhances inflammation in colitis mice. Nevertheless, 4-octyl itaconate (4-OI), a derivative of itaconate, retards the effects [17]. Besides, Acod1 overexpression and 4-OI supplementation are able to protect against inflammation of microglia and facilitate functional recovery in mice with spinal cord injury [18]. Itaconate mediated by Acod1 limits pulmonary fibrosis [19], blocks pulmonary Brucella infection [20] and prevents abdominal aortic aneurysm formation in mice [21]. However, the effects of Acod1 and itaconate and the potential molecular mechanisms are still unclear in obesity-induced pulmonary microvascular endotheliopathy.

Nuclear factor erythroid 2-like 2 (Nrf2) is an antioxidant transcriptional activator. A previous study has shown that Acod1/Itaconate represses oxidative injury and inflammation during liver ischemia-reperfusion by activating Nrf2 in mice or hepatocytes [22]. In addition, hepatic miR-144 targets Acod1, causing the reduction of itaconate and the consumption of TCA metabolite fumarate, thereby inhibiting Nrf2 activity, which triggers an impaired antioxidant response in obese mice and humans [23]. It is thus clear that Nrf2 activation is required during treatment of Acod1 and itaconate for pulmonary microvascular endotheliopathy caused by obesity.

In this study, our aim is to explore the effects and potential mechanisms of Acod1 and 4-OI on free fatty acid (FFA) induced mouse PMVEC injury, inflammation and mitochondrial oxidative stress.

## Materials and methods

### Mice

Six-week-old male C57BL/6J mice were fed with a standard diet (chow) or a high-fat diet (HFD) for 29 weeks. All mouse body weights were measured once a week. The mice were euthanized with isoflurane anesthesia and exsanguinated from inferior vena cava. The lung, liver, and adipose tissues were collected and photographed. The lung tissues were used for subsequent PMVEC isolation immediately. The animal protocol was approved by the Ethics Committee of Animal Research at the Henan Provincial Institute for Food and Drug Control (No. 232300421122-1).

### Isolation and identification of mouse PMVECs

The collected lung tissues were cut into small pieces and grinded to obtain cell suspension. Subsequently, CD45^−^ and CD31^+^ PMVECs selected by the CD45 and CD31 magnetic beads (Miltenyi Biotec, Gladbach, Germany; 10 μL beads/10^7^ cells) were inoculated onto Dulbecco’s Modified Eagle Medium/Nutrient Mixture F-12 (DMEM/F12) medium (Biosharp, Hefei, China) supplemented with 10% fetal bovine serum (FBS) in 5% CO_2_ at 37℃. Immunofluorescence was used to detect the expression of CD31 and vascular endothelial cadherin (VE-cadherin) in the PMVECs. Briefly, the PMVECs were fixed in 4% paraformaldehyde (Sinoreagent, Shanghai, China) and permeabilized in 0.1% Triton X-100 (Beyotime Biotech, Shanghai, China). The PMVECs were incubated with anti-rabbit CD31 (1:100; Abclonal, Shanghai, China) and VE-cadherin (Abclonal, Shanghai, China) at 4℃ overnight. Then, the cells were incubated with Cy3-labeled goat anti-rabbit IgG (1:1000; Abcam, Cambridge, UK) at room temperature for one hour. 2-(4-amidinophenyl) 6-indolecarbamidine dihydrochloride (DAPI; Aladdin, Shanghai, China) was used for counterstaining nuclei. The images were captured using a fluorescence microscope (Olympus, Japan).

### mRNA sequencing

Total RNA was extracted from the PMVECs of chow and HFD mice using the TRIpure (BioTeke, Beijing, China) and subsequently used for RNA-seq. The data were analyzed using Illumina sequencing platform. The DEGs were determined by |log_2_ fold change| ≥ 1 and *p* ≤ 0.05. Furthermore, Reactome, BioCyc and PANTHER, KEGG and GO pathway analyses were conducted to analyze these DEGs.

### FFA content

The lung tissues were washed with physiological saline, and the surface moisture was absorbed using absorbent paper. Next, the tissue homogenate was obtained in an ice bath using extract liquid (10 mL/g) and centrifuged at 8000 rpm for 10 min at 4℃ to get the supernatant. The protein contents were measured by the BCA Protein Assay Kit (Beyotime Biotech, Shanghai, China). The levels of FFA in mouse plasma and lungs were determined using the FFA Content Assay Kit (Solarbio, Beijing, China).

### Cell treatment

For FFA treatment, the PMVECs were obtained from the male C57BL/6J mice (8–10 weeks old) by CD45^−^ and CD31^+^ selection using the above methods. These cells were treated with 500 μM FFAs containing 25% sodium-palmitic acid (Macklin, Shanghai, China), 25% sodium-oleic acid (Macklin, Shanghai, China), 25% arachidonic acid (Yuanye, Shanghai, China) and 25% stearic acid (Macklin, Shanghai, China) [24] for 12, 24, 36 and 48 h. Next, the cells were harvested for Acod1 RNA or protein detection. The subsequent experiments were performed at FFA treatment for 36 h.

For cell transfection, the PMVECs were cultured in 5% CO_2_ at 37℃. Acod1 overexpressed plasmid or Nrf2 siRNA (si-Nrf2) was transfected into the cells using LipofectamineTM 3000 Reagent (Invitrogen, Carlsbad, CA, USA). Blank plasmids or negative control siRNA (si-NC) were used as controls. For 4-OI administration, the cells were treated using 250 μM 4-OI (Yuanye, Shanghai, China) or vehicle control. FFA treatment was performed after transfection for 36 h or 4-OI administration for 2 h. The efficiency of cell transfection was determined by real-time PCR and western blot after transfection for 36 h.

### Cell viability

The PMVECs (5 × 10^3^/mL) were cultured in 96-well plates in 5% CO_2_ at 37℃ (5 × 10^3^/well). Next, CCK-8 (10 μL) was added and cultured for 2 h. The data were recorded at OD 450 nm by a microplate reader (BioTek, USA). The PMVEC viability was assessed by Cell-Counting-Kit-8 (CCK-8; KeyGene Biotech, Nanjing, China).

### Succinate dehydrogenase (SDH) and caspase 3 activity

The SDH activities of the cells were detected using the SDH Activity Assay Kit (Solarbio, Beijing, China). The Caspase 3 Activity Assay Kit (Beyotime Biotech, Shanghai, China) was applied to determine caspase 3 enzyme activities in the cells.

### MitoSOX red staining

The levels of mitochondrial reactive oxygen species (ROS) were determined using MitoSOX Red (Maokang Biotech, Shanghai, China) staining, thereby reflecting the levels of oxidative stress in PMVECs. Briefly, the cells were incubated with 5 μM MitoSOX Red for 10 min at 37℃. Fluorescent intensity was measured using an inverted phase contrast microscope (Olympus, Japan).

### Rhodamine 123

Rhodamine 123 (Macklin, Shanghai, China) was used to estimate mitochondrial membrane potential (MMP) in PMVECs. Briefly, the cells were stained with 5 μM Rhodamine 123 and incubated in 5% CO_2_ for 30 min at 37℃. The cells were washed three times using PBS. Fluorescent intensity was measured using an inverted phase contrast microscope (Olympus, Japan).

### Immunofluorescence

Endothelial activation marker endothelin-1 (ET-1) expression in the PMVECs was evaluated by immunofluorescence assay. As described previously, the PMVECs were fixed, permeabilized, and then incubated with anti-mouse ET-1 (1:100; Bioss, Beijing, China) at 4℃ overnight. Next, the cells were incubated with Cy3-labeled goat anti-mouse IgG (1:200; Abcam, Cambridge, UK) at room temperature for one hour. The nuclei were counterstained by DAPI (Aladdin, Shanghai, China). The images were captured using a fluorescence microscope (Olympus, Japan).

### Real-time PCR

Total RNA was extracted from the PMVECs using TRIpure reagent (BioTeke, Beijing, China). BeyoRT II M-MLV Reverse Transcriptase (Beyotime, Shanghai, China) was used to obtain the cDNA. The DNA was analyzed by real-time PCR with SYBR Green I (Solarbio, Beijing, China). The primers were synthesized by Generalbiol (Chuzhou, China). GAPDH was used as an internal control. The sequences were as follows: Acod1 forward: 5’-ATGGTATCATTCGGAGGAG-3’; Acod1 reverse: 5’-CAAACAGTGCTGGAGGTG-3’; ET-1 forward: 5’-GTCTTGGGAGCCGAACT-3’; ET-1 reverse: 5’-TCTAACTGCCTGGTCTGTG-3’; ICAM-1 forward: 5’-CTGGCAGCAAGTAGGCA-3’; ICAM-1 reverse: 5’-CTGGCGGCTCAGTATCT-3’; MCP-1 forward: 5’-GCCTGCTGTTCACAGTTGCC-3’; MCP-1 reverse: 5’-CTGGACCCATTCCTTCTTGG-3’; IL-6 forward: 5’-TAACAGATAAGCTGGAGTC-3’; IL-6 reverse: 5’-TAGGTTTGCCGAGTAGA-3’; Nrf2 forward: 5’-GTGCTCCTATGCGTGAA-3’; Nrf2 reverse: 5’-GCGGCTTGAATGTTTGT-3’.

### Western blot

The PMVECs were lysed with cell lysates and then separated by SDS-polyacrylamide gel electrophoresis (Solarbio, Beijing, China) and transferred onto polyvinylidenedifluoride membranes (Millipore, USA). The samples were incubated with anti-rabbit Acod1 antibody (1:1000; Abcam, Shanghai, China) or anti-rabbit Nrf2 antibody (1:1000; ABclonal, Shanghai, China) at 4℃ overnight. Anti-rabbit Histone H3 (1:5000; Gene Tex, USA) or anti-mouse GAPDH (1:10000; Proteintech, Wuhan, China) were used as the controls. Subsequently, these cells were subjected to incubation with HRP-conjugated goat anti-rabbit IgG (1:3000; Solarbio, Beijing, China) or HRP-conjugated goat anti-mouse IgG (1:3000; Solarbio, Beijing, China) at 37℃ for one hour. The proteins were visualized by enhanced chemiluminescence (Solarbio, Beijing, China).

### Statistical analysis

GraphPad Prism software was applied to analyze the data, and the results were expressed as mean ± SD. The differences between two groups or multiple groups were assessed via unpaired t test and One-way ANOVA, respectively. The data of chow and HFD mouse weight were analyzed via Two-way repeated measures ANOVA. *p* values less than 0.05 were considered statistically significant.

## Results

### The DEGs between chow and HFD mouse PMVECs were identified by mRNA-seq

**Fig. 1:**
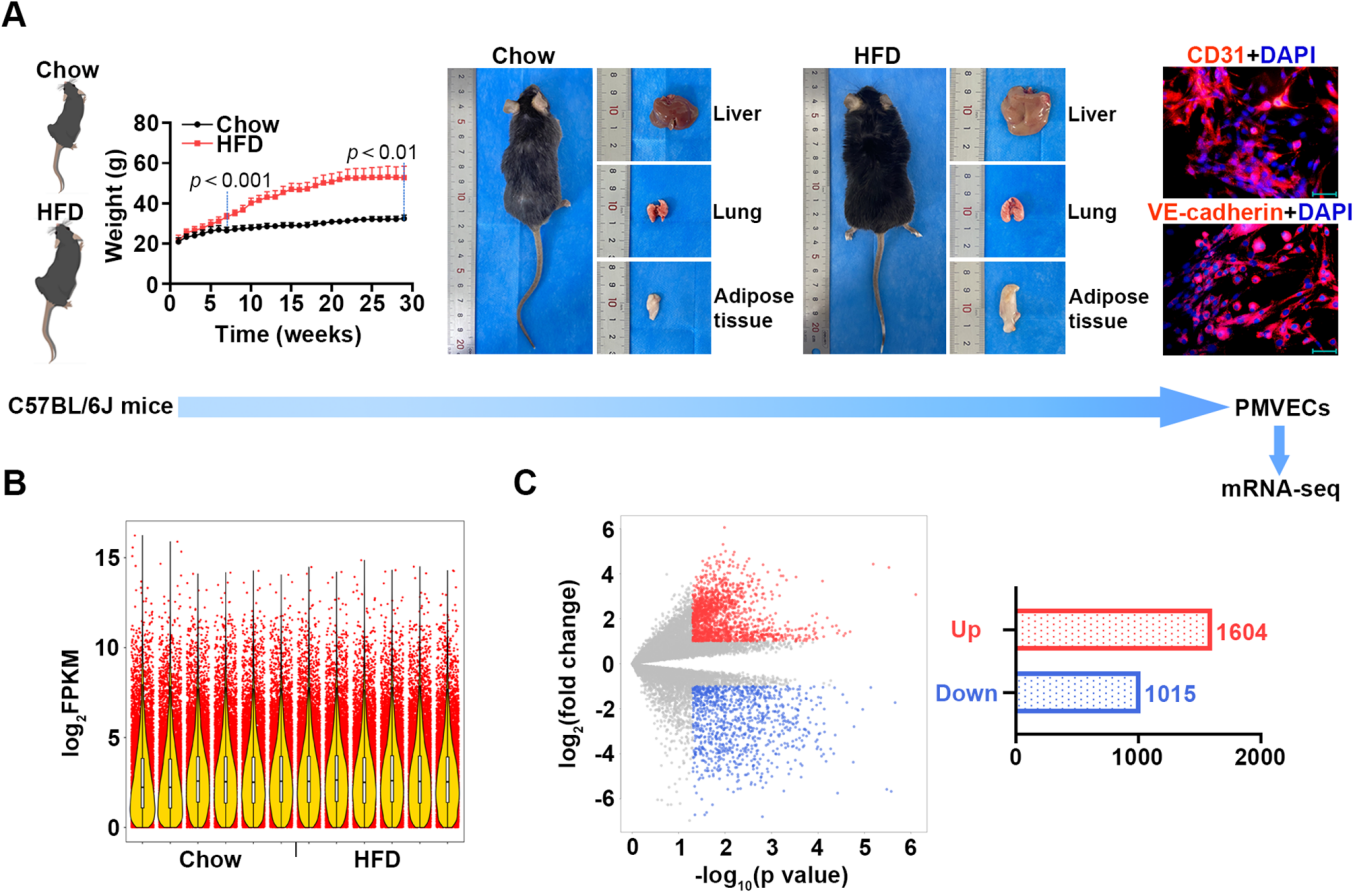
mRNA-seq analysis reveals the DEGs between the PMVECs of chow and HFD mice. (**A**) A flow chart describing the experiments before mRNA-seq. The mice were weighed during chow or HFD feeding (*n* = 6). Representative images of the mice and lung, liver and adipose tissues after chow or HFD feeding were shown. The primary PMVECs isolated from the mice were identified by immunofluorescent staining for CD31 and VE-cadherin (blue: DAPI; red: CD31 and VE-cadherin; Scale bar = 50 μm). The mouse drawings are from SciDraw (https://zenodo.org/record/3925997). (**B**) A violin plot of the log_2_FPKM value of all mRNAs within each sample. (**C**) A volcano plot showing the DEGs between chow and HFD samples (|log2 fold change| ≥ 1, *p* ≤ 0.05; Up-regulated gene counts = 1604; Down-regulated gene counts = 1015)

**Fig. 2:**
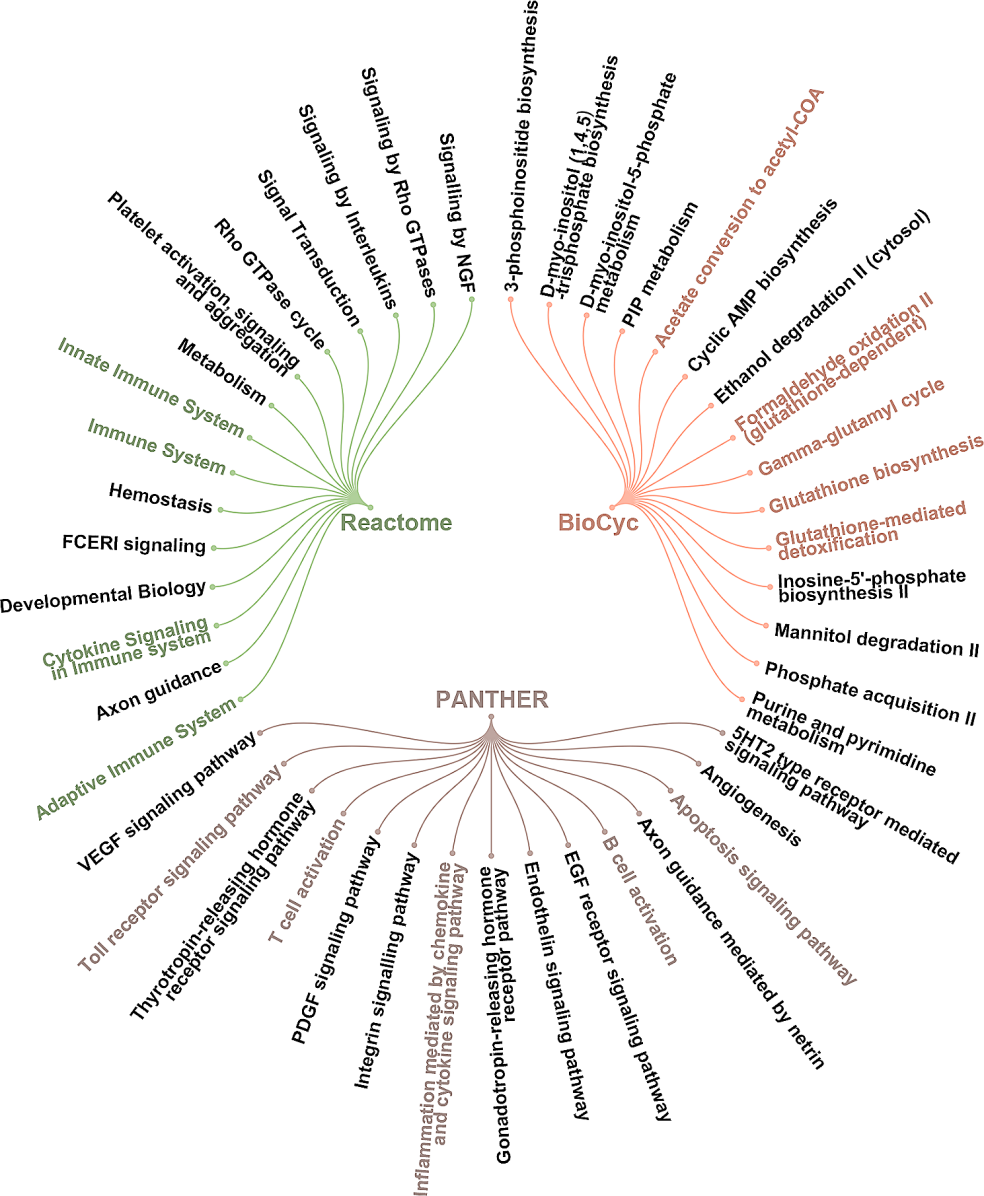
The circle diagram showing the top 15 significant terms of Reactome, BioCyc and PANTHER for the DEGs between chow and HFD samples

As shown in Fig. 1A, the chow and HFD mice were weighed during feeding for 29 weeks. The weight of HFD mice increased rapidly and significantly compared to chow mice after feeding for 7 weeks. Furthermore, HFD mice appeared hepatic steatosis, lung and epididymal fat volume increases. The isolated PMVECs were identified and screened through immunofluorescent staining for CD31 and VE-cadherin (Fig. 1A). The screened PMVECs were used to perform mRNA-seq. A violin plot showed the distribution of log_2_FPKM value across all the samples (Fig. 1B). The DEGs between the chow and HFD samples were displayed in a volcano plot. There were 1604 up-regulated and 1015 down-regulated DEGs (Fig. 1C; |log_2_ fold change| ≥ 1, *p* ≤ 0.05). The pathways enriched by the DEGs were analyzed via Reactome, BioCyc and PANTHER. Each of the top 15 significant terms was shown in Fig. 2, indicating that the DEGs were mainly enriched in immune, inflammation, apoptosis, and TCA associated pathways (Fig. 2). The Top 30 significant enriched KEGG and GO pathway terms were displayed in Figure S1, which showed that the DEGs were related to immune response, inflammation, and so on (Figure S1). Interestingly, we found that the fourth most significantly down-regulated differential gene was Acod1, which was reported to be closely linked with those pathways (Table 1). Therefore, Acod1 was identified and served as the main research object in this study.

**Table 1:** The top 10 significantly upregulated and downregulated DEGs in HFD mice

Ensemble ID	Log_2_FC	Regulation	Symbol	Description
ENSMUSG00000025330	-6.80	down	Padi4	peptidyl arginine deiminase 4
ENSMUSG00000058755	-6.71	down	Osm	oncostatin M
ENSMUSG00000018930	-6.71	down	Ccl4	C-C motif chemokine ligand 4
ENSMUSG00000022126	-6.61	down	Acod1	aconitate decarboxylase 1
ENSMUSG00000022584	-6.26	down	Ly6c2	lymphocyte antigen 6 family memberC2
ENSMUSG00000022097	-6.02	down	Sftpc	surfactant protein C
ENSMUSG00000018924	-5.75	down	Alox15	arachidonate 15-lipoxygenase
ENSMUSG00000005824	-5.74	down	Tnfsf14	tumor necrosis factor superfamily member 14
ENSMUSG00000026581	-5.70	down	Sell	selectin L
ENSMUSG00000026414	-5.67	down	Tnnt2	troponin T2, cardiac type
ENSMUSG00000079092	6.06	up	Prl2c2	prolactin family 2, subfamily c, member 2
ENSMUSG00000021214	5.30	up	Akr1c18	aldo-keto reductase family 1, member C18
ENSMUSG00000105867	5.00	up	Gm42517	transmembrane 217, retrotransposed
ENSMUSG00000056457	4.96	up	Prl2c3	prolactin family 2, subfamily c, member 3
ENSMUSG00000037334	4.86	up	H2-M1	histocompatibility 2, M region locus 1
ENSMUSG00000024743	4.84	up	Syt7	synaptotagmin 7
ENSMUSG00000074934	4.76	up	Grem1	gremlin 1, DAN family BMP antagonist
ENSMUSG00000029659	4.69	up	Medag	mesenteric estrogen dependent adipogenesis
ENSMUSG00000042717	4.67	up	Ppp1r3a	protein phosphatase 1 regulatory subunit 3 A
ENSMUSG00000019732	4.62	up	Calr3	calreticulin 3

### Acod1 was down-regulated in HFD mice and FFA-treated PMVECs

**Fig. 3:**
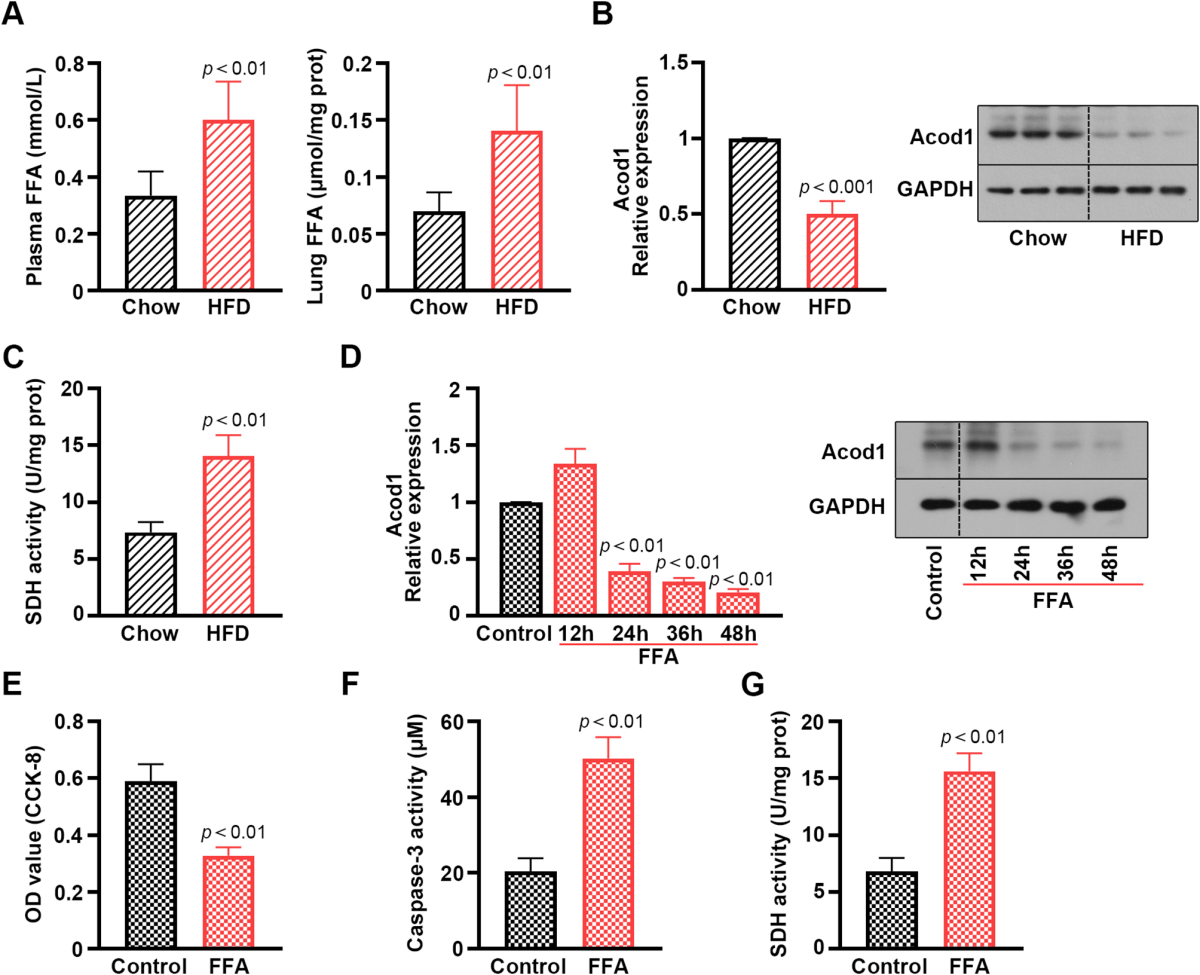
The FFA contents and SDH activity are detected, and Acod1 expression is verified in HFD mice. Besides, FFA prevents cell viability and promotes caspase-3 and SDH activities in mouse PMVECs. (**A**) The contents of FFA were increased in the HFD mouse plasma and lung, respectively, compared with the chow mice. (**B**) The expression of Acod1 was down-regulated in the HFD mouse PMVECs. (**C**) SDH activity was elevated in the HFD mouse PMVECs. (**D**) Acod1 expression was detected in the PMVECs at different time points after 500 μM FFA treatment. (**E**) Cell viability was measured in the PMVECs stimulated by FFA for 36 h. (**F**) The activities of caspase-3 and (**G**) SDH were detected in the PMVECs by the corresponding kits

FFA contents were augmented in HFD mouse plasma and lung tissues (Fig. 3A). Down-regulated Acod1 expression was verified by real-time PCR and western blot (Fig. 3B). SDH activity was elevated in HFD mice (Fig. 3C). Additionally, FFA inhibited Acod1 expression in PMVECs as time went on (Fig. 3D). FFA induced decreased cell viability as well as increased caspase 3 and SDH activities in PMVECs (Fig. 3E-G). These results revealed a down-regulated Acod1 in HFD mice and FFA-treated PMVECs and suggested that FFA accelerated cell injury in PMVECs.

### Acod1 blocked FFA-induced cell injury, inflammation and mitochondrial oxidative stress in mouse PMVECs

**Fig. 4:**
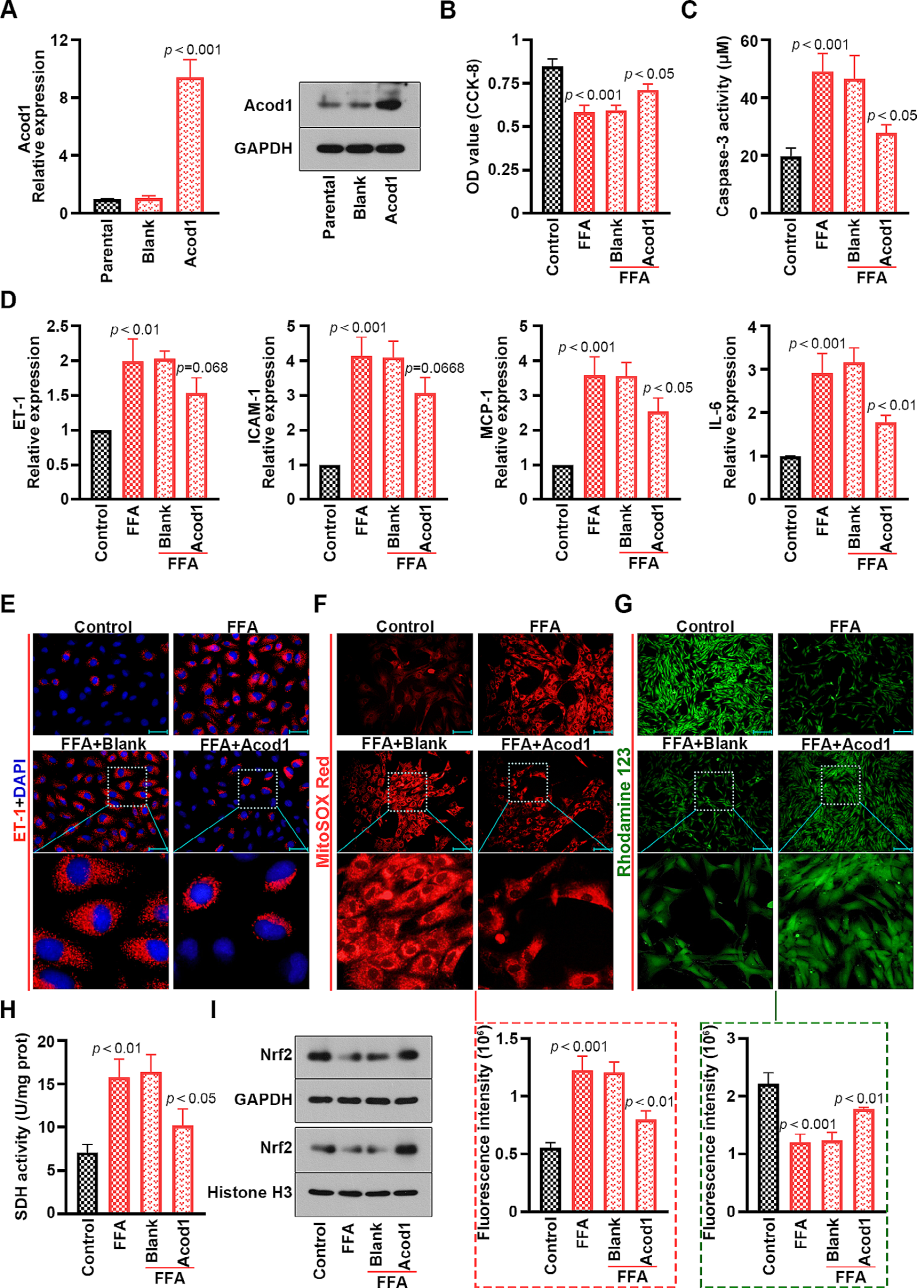
Acod1 inhibits FFA-induced decline of cell viability and increase of apoptosis, inflammation and mitochondrial oxidative stress in mouse PMVECs. (**A**) Acod1 expression was examined by real-time PCR and western blot in the PMVECs. (**B**) The viability of PMVECs. (**C**) Activity of caspase-3 in the PMVECs. (**D**) The relative expression levels of endothelial activation marker ET-1, and inflammatory marker ICAM-1, MCP-1 and IL-6 in the PMVECs. (**E**) The expression of ET-1 in the PMVECs was determined by immunofluorescence staining (blue: DAPI; red: ET-1; Scale bar = 50 μm). (**F**) The mitochondria of the PMVECs were stained by MitoSOX Red (red; Scale bar = 100 μm) and (**G**) Rhodamine 123 (green; Scale bar = 200 μm). (**H**) The SDH activity of the PMVECs. (**I**) The Nrf2 protein levels in the PMVECs and the nucleus

The Acod1 overexpressed plasmid was established, and the expression was verified by real-time PCR and western blot (Fig. 4A). It was found that overexpressing Acod1 reversed FFA-stimulated the reduction of cell viability and increase of cell apoptosis in PMVECs (Fig. 4B and C). The levels of endothelial activation marker ET-1 as well as inflammatory marker ICAM-1, MCP-1 and IL-6 were enhanced in FFA-treated PMVECs. However, the overexpression of Acod1 ameliorated these effects (Fig. 4D). The effects on ET-1 expression were also determined by immunofluorescence (Fig. 4E). We observed that FFA promoted ROS production, whereas Acod1 suppressed the effect by MitoSOX Red staining (Fig. 4F). Moreover, the results of Rhodamine 123 staining illustrated that Acod1 restored the MMP collapse induced by FFA (Fig. 4G). Increased SDH activity stimulated by FFA was reduced by Acod1 overexpression (Fig. 4H). We demonstrated that Acod1 reversed the inhibition of Nrf2 protein levels caused by FFA in the PMVECs and cell nucleus (Fig. 4I). The results confirmed that Acod1 protected mouse PMVECs from FFA-triggered cell injury, inflammation and mitochondrial oxidative stress.

### 4-OI prevented FFA-induced cell injury, inflammation and mitochondrial oxidative stress in mouse PMVECs

**Fig. 5:**
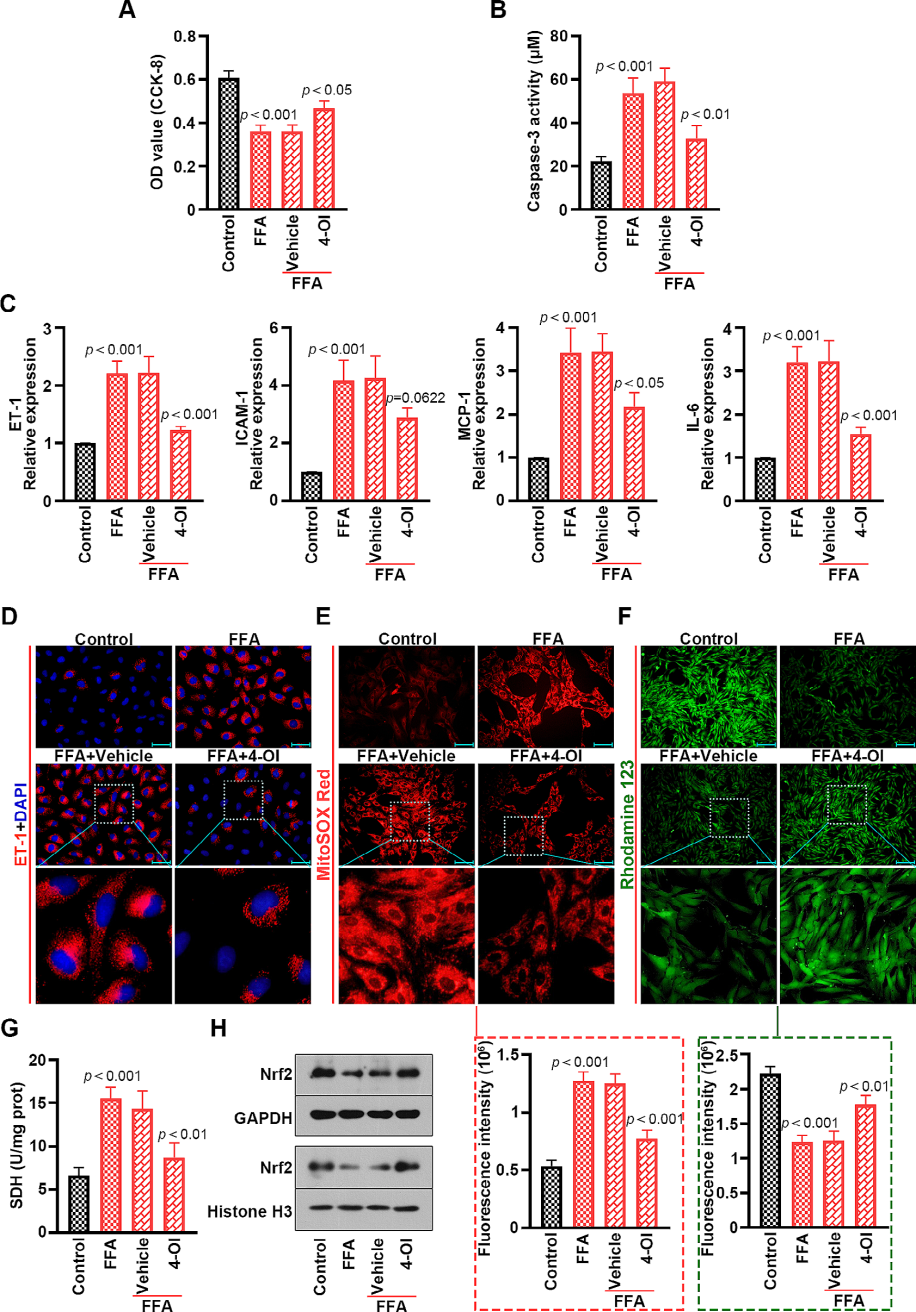
4-OI represses FFA-induced decrease of cell viability and increase of apoptosis, inflammation and mitochondrial oxidative stress in mouse PMVECs. (**A**) The viability of PMVECs. (**B**) Activity of caspase-3 in the PMVECs. (**C**) The relative expression levels of ET-1, ICAM-1, MCP-1 and IL-6 in the PMVECs. (**D**) The expression of ET-1 in the PMVECs was determined by immunofluorescence staining (blue: DAPI; red: ET-1; Scale bar = 50 μm). (**E**) The mitochondria of the PMVECs were stained by MitoSOX Red (red; Scale bar = 100 μm) and (**F**) Rhodamine 123 (green; Scale bar = 200 μm). (**G**) The SDH activity of the PMVECs. (**H**) The Nrf2 protein levels in the PMVECs and the nucleus

Consistently with the effects of Acod1 overexpression, 4-OI reversed the effects of FFA on cell viability and apoptosis in PMVECs (Fig. 5A and B). Besides, increased ET-1, ICAM-1, MCP-1 and IL-6 levels induced by FFA were restrained by 4-OI (Fig. 5C and D). The results of MitoSOX Red and Rhodamine 123 staining uncovered that 4-OI effectively repressed the ascending ROS contents and descending MMP caused by FFA (Fig. 5E and F). 4-OI alleviated FFA-activated SDH activity elevation (Fig. 5G). The western blot assay showed that 4-OI restored the decreased protein levels of Nrf2 triggered by FFA in the PMVECs and cell nucleus (Fig. 5H). Accordingly, 4-OI exerted a protective role in FFA-treated PMVECs.

### Acod1 and 4-OI protected mouse PMVECs from FFA-induced injury, inflammation and mitochondrial oxidative stress through Nrf2

**Fig. 6:**
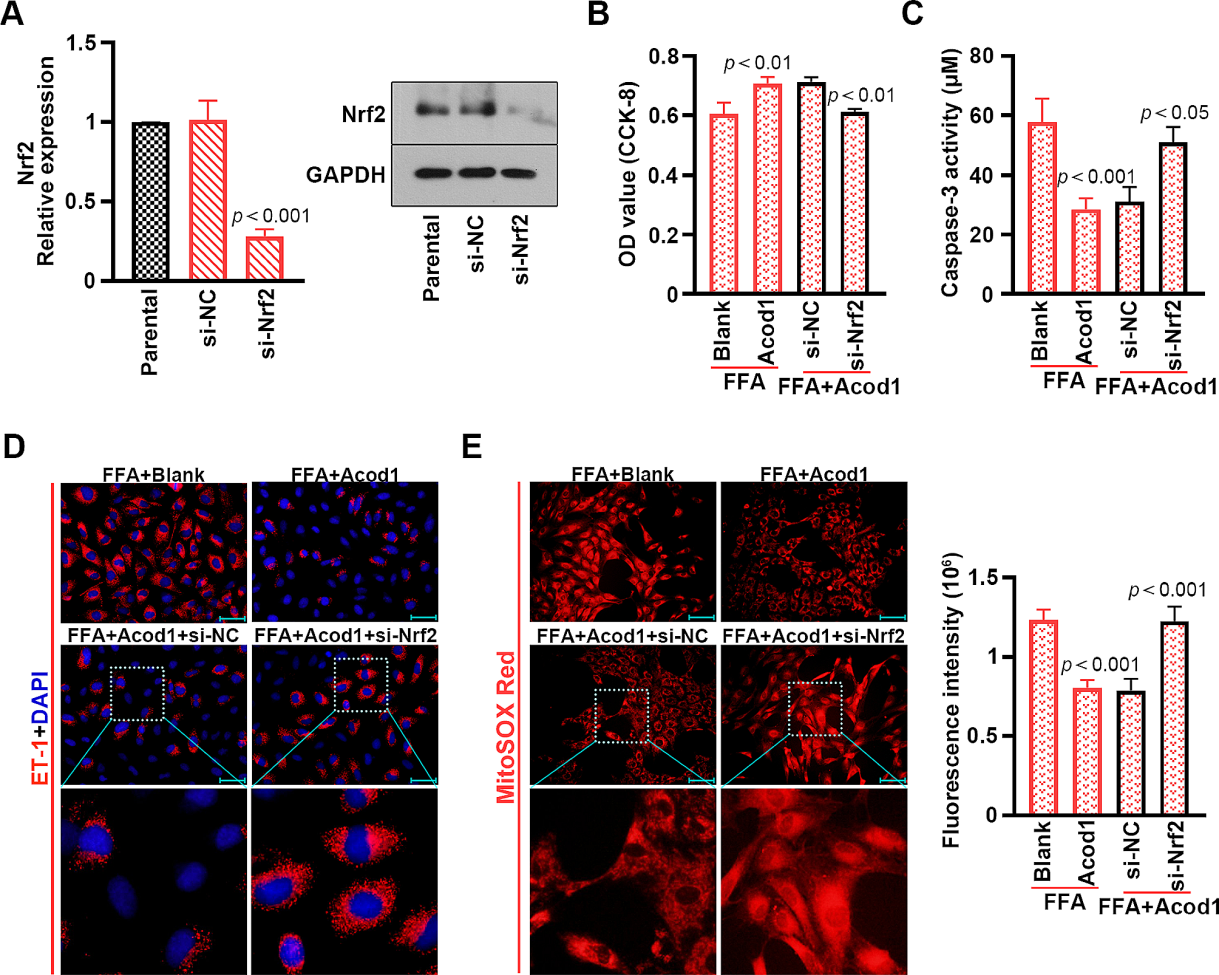
Silencing Nrf2 blocks the effects of Acod1 on FFA-stimulated cell viability, apoptosis, inflammation and mitochondrial oxidative stress in mouse PMVECs. (**A**) The mRNA and protein levels of Nrf2. (**B**) The viability of PMVECs. (**C**) Activity of caspase-3 in the PMVECs. (**D**) Immunofluorescence staining to show ET-1 expression (blue: DAPI; red: ET-1; Scale bar = 50 μm). (**E**) Fluorescence intensity of MitoSOX Red (red; Scale bar = 100 μm)

**Fig. 7:**
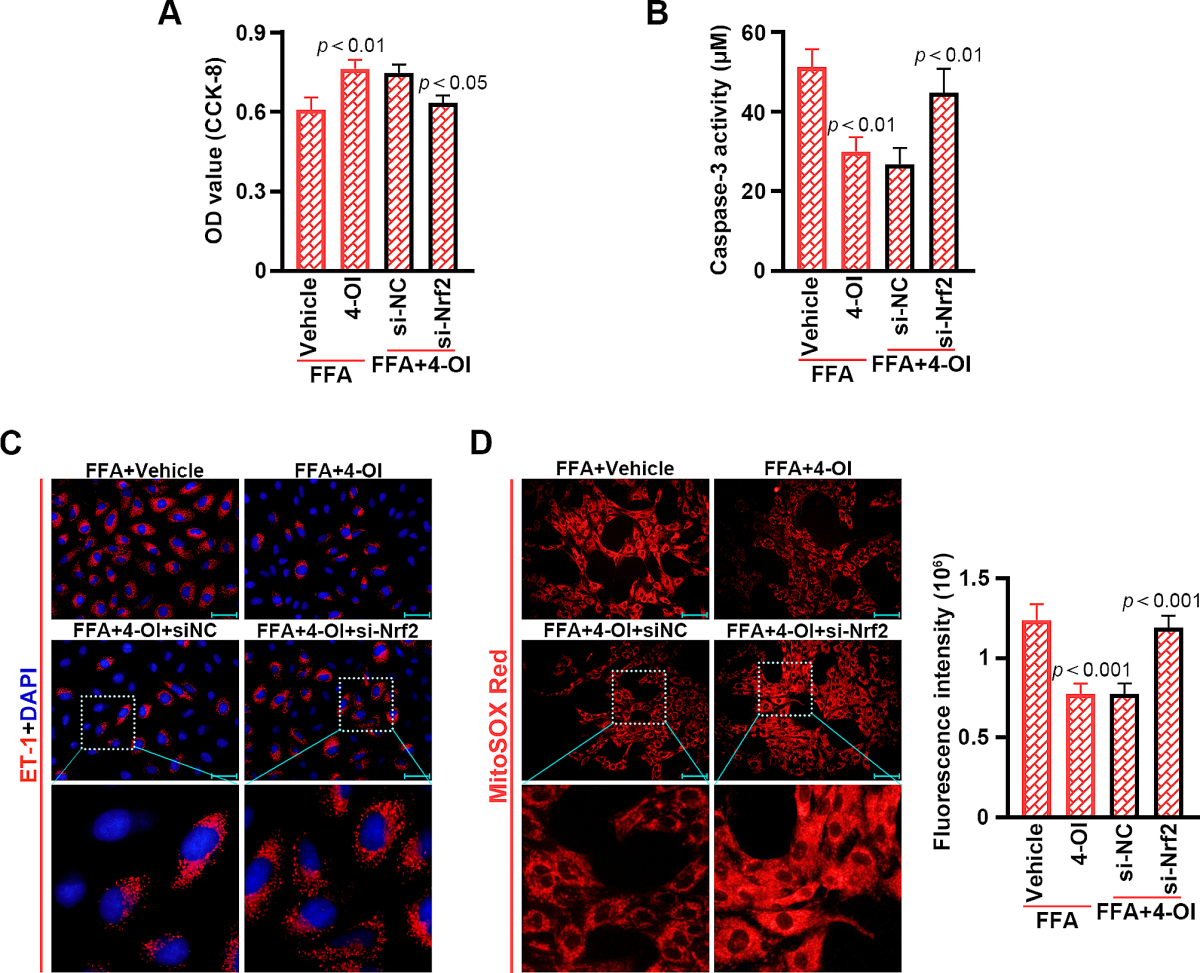
Silencing Nrf2 blocks the effects of 4-OI on FFA-stimulated cell viability, apoptosis, inflammation and mitochondrial oxidative stress in mouse PMVECs. (**A**) The viability of PMVECs. (**B**) Activity of caspase-3 in the PMVECs. (**C**) Immunofluorescence staining to show ET-1 expression (blue: DAPI; red: ET-1; Scale bar = 50 μm). (**D**) Fluorescence intensity of MitoSOX Red (red; Scale bar = 100 μm)

Nrf2 expression was down-regulated in the PMVECs transfected with the Nrf2 siRNA pool (Fig. 6A). Silencing Nrf2 reversed the effects of Acod1 overexpression on cell viability, apoptosis, inflammation and mitochondrial oxidative stress in FFA-treated PMVECs (Fig. 6B-E). Consistently, 4-OI improved the FFA-stimulated effects, which were blocked by Nrf2 down-regulation (Fig. 7A-D). These results indicated that Nrf2 was required for Acod1 and 4-OI-ameliorated cell injury, inflammation and mitochondrial oxidative stress in the FFA-stimulated PMVECs.

## Discussion

Obesity is capable of inducing microvascular endothelial dysfunction [8]. In the current study, DEGs were identified in HFD and chow mouse PMVECs by mRNA-seq, and a significantly down-regulated Acod1 was exposed. Administration of FFA is widely known to trigger overproduction of ROS, inflammatory response, and endothelial cell dysfunction [25]. Herein, we used FFA to induce cell injury and inflammation, which were reversed by Acod1 overexpression and 4-OI administration in the PMVECs. Moreover, Acod1 and 4-OI restored FFA-caused excessive production of ROS and MMP loss to varying degrees. Importantly, the effects of Acod1 and 4-OI were dependent on the regulation of Nrf2 levels. Our findings suggested that Acod1/Itaconate axis might be an effective therapeutic treatment for mice with obesity-induced pulmonary microvascular endotheliopathy by activating Nrf2.

Acod1 is initially discovered in lipopolysaccharide-induced macrophages [15]. Acod1 serves as a cis-aconitate decarboxylase that catalyzes the production of itaconic acid, which links metabolism with immune response [16, 26, 27]. Itaconate is a metabolic intermediate of the TCA cycle and functions as a regulator of inflammatory responses [28, 29]. These findings have suggested that Acod1 and itaconate participate in immune response, inflammatory and TCA cycle pathways. In our study, we analyzed the data of mRNA-seq by Reactome, BioCyc and PANTHER, KEGG and GO pathway enrichment analysis and found that the DEGs between HFD and chow mice were linked with the immune system mainly. Besides, the DEGs were involved in the TCA cycle-related pathways, such as acetate conversion to acetyl-CoA, gamma-glutamyl cycle, and glutathione-involved pathways. Moreover, Acod1 was down-regulated markedly in HFD-fed mouse PMVECs. It was preliminary demonstrated that Acod1 might play a critical role in obesity-induced pulmonary microvascular endotheliopathy by regulating immune, inflammation responses and the TCA cycle.

Acod1 silencing inhibits antibacterial activity during microbial infection [27]. Acod1 deficiency promotes pulmonary *Brucella* infection in mice [20]. Acod1 induced by hemeoxygenase-1 or carbon monoxide constricts LPS-mediated sepsis and the production of pro-inflammatory cytokines [30]. Additionally, Acod1 has an inhibitory role in mouse cerebral ischemia-reperfusion injury [31]. These findings uncover the protective effect of Acod1 against injury in diseases. Consistently, Acod1 overexpression protected the mouse PMVECs from FFA-induced injury, including the decrease of cell viability and the increase of cell apoptosis, inflammation and oxidative stress. Itaconate produced by Acod1 catalysis triggered the consistent effects.

Previous studies have indicated that Acod1/Itaconate axis exerts protective roles in diseases by activating Nrf2. Nrf2 is a transcription factor that has the function of resisting injury, inflammation and oxidative stress [32]. Liver ischemia-reperfusion causes inflammation and oxidative stress, which is reversed by Acod1/Itaconate via advancing Nrf2-mediated anti-oxidative reaction in hepatocytes [22]. Besides, Acod1 stimulates the Nrf2/heme oxygenase-1 pathway and suppresses ROS activation pathways, thereby protecting mice against concanavalin A-caused liver injury [33]. Acod1/Itaconate also protects renal cells from oxidative stress induced by ischemia-reperfusion injury and acute kidney injury by promoting the Nrf2 pathway [34]. Itaconate diminishes sepsis-induced acute lung injury through inhibiting ferroptosis of macrophages, which requires the participation of Nrf2 [35]. Itaconate alkylates Kelch-like ECH-associated protein 1 thereby stimulating Nrf2 to exert anti-oxidant and anti-inflammatory capacities in mouse and human macrophages [14]. Our work provided evidence that the decreased expression of Nrf2 in the nucleus and cytoplasm induced by FFA was restored after Acod1 overexpression and 4-OI administration. Silencing Nrf2 mitigated the protective effects of Acod1 and itaconate in FFA-treated PMVECs, indicating Acod1/itaconate depended on Nrf2 to exhibit the effects.

In summary, our study identified differentially expressed Acod1 in obese mouse PMVECs. We revealed that Acod1 and itaconate protect mouse PMVECs from FFA-induced injury, oxidative stress and inflammation via activating Nrf2. The study provided promising treatment strategies for obesity-caused pulmonary vascular disease.

### Electronic supplementary material

Below is the link to the electronic supplementary material.

Supplementary Material 1

Supplementary Material 2

Supplementary Material 3

Supplementary Material 4

Supplementary Material 5

Supplementary Material 6

Supplementary Material 7

Supplementary Material 8

Supplementary Material 9

Supplementary Material 10

Supplementary Material 11

Supplementary Material 12

Supplementary Material 13

Supplementary Material 14

Supplementary Material 15

Supplementary Material 16

Supplementary Material 17

## Data Availability

No datasets were generated or analysed during the current study.
