# Supplementary figures and images for: Acod1/itaconate activates Nrf2 in pulmonary microvascular endothelial cells to protect against the obesity-induced pulmonary microvascular endotheliopathy
Source: Respir Res. 2024 May 10;25:205. doi: 10.1186/s12931-024-02827-w (PMC11088094; doi:10.1186/s12931-024-02827-w)

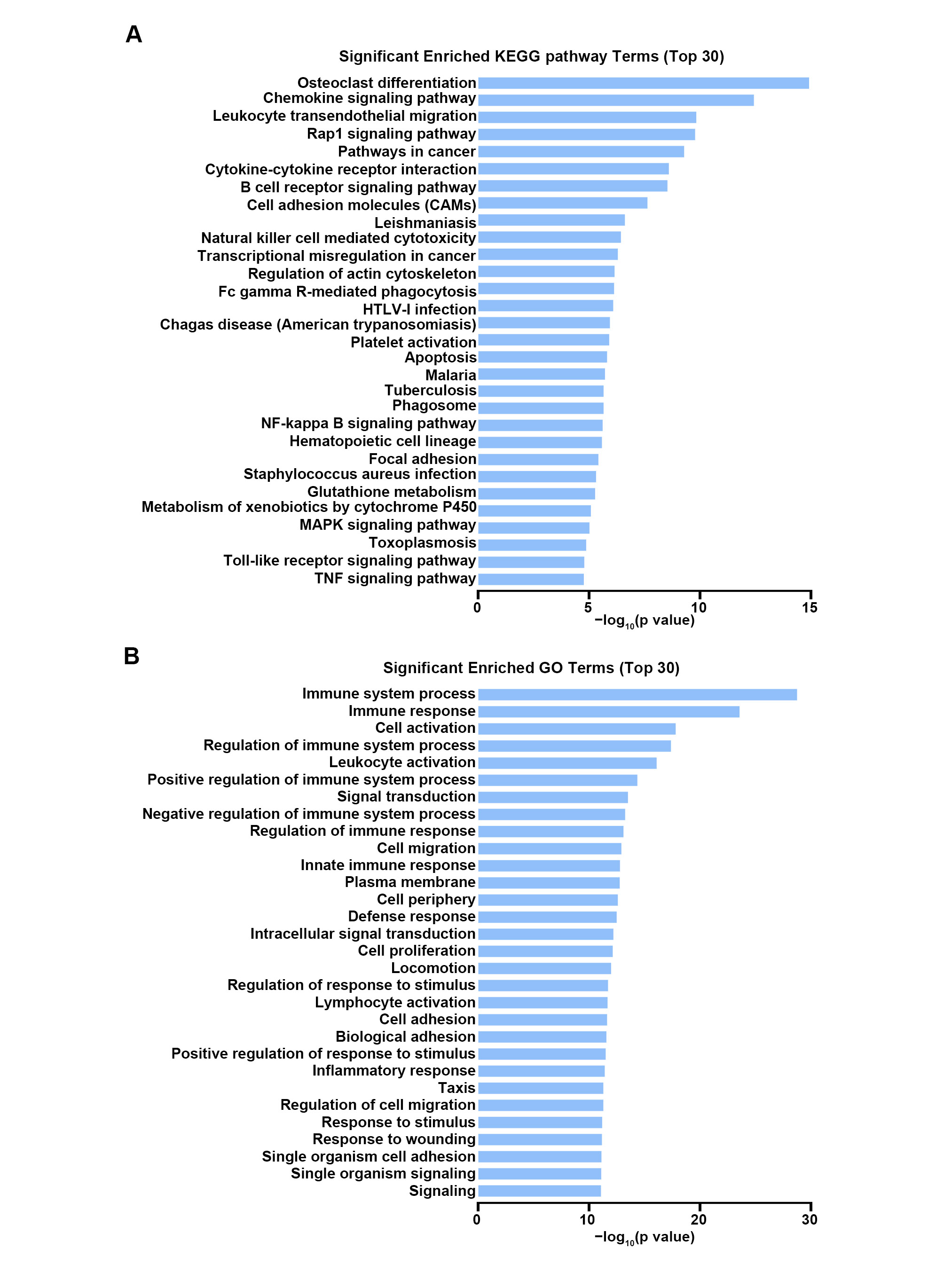

Supplement: Supplementary file 1 — Supplementary Material 1 [file 12931_2024_2827_MOESM1_ESM.jpg]

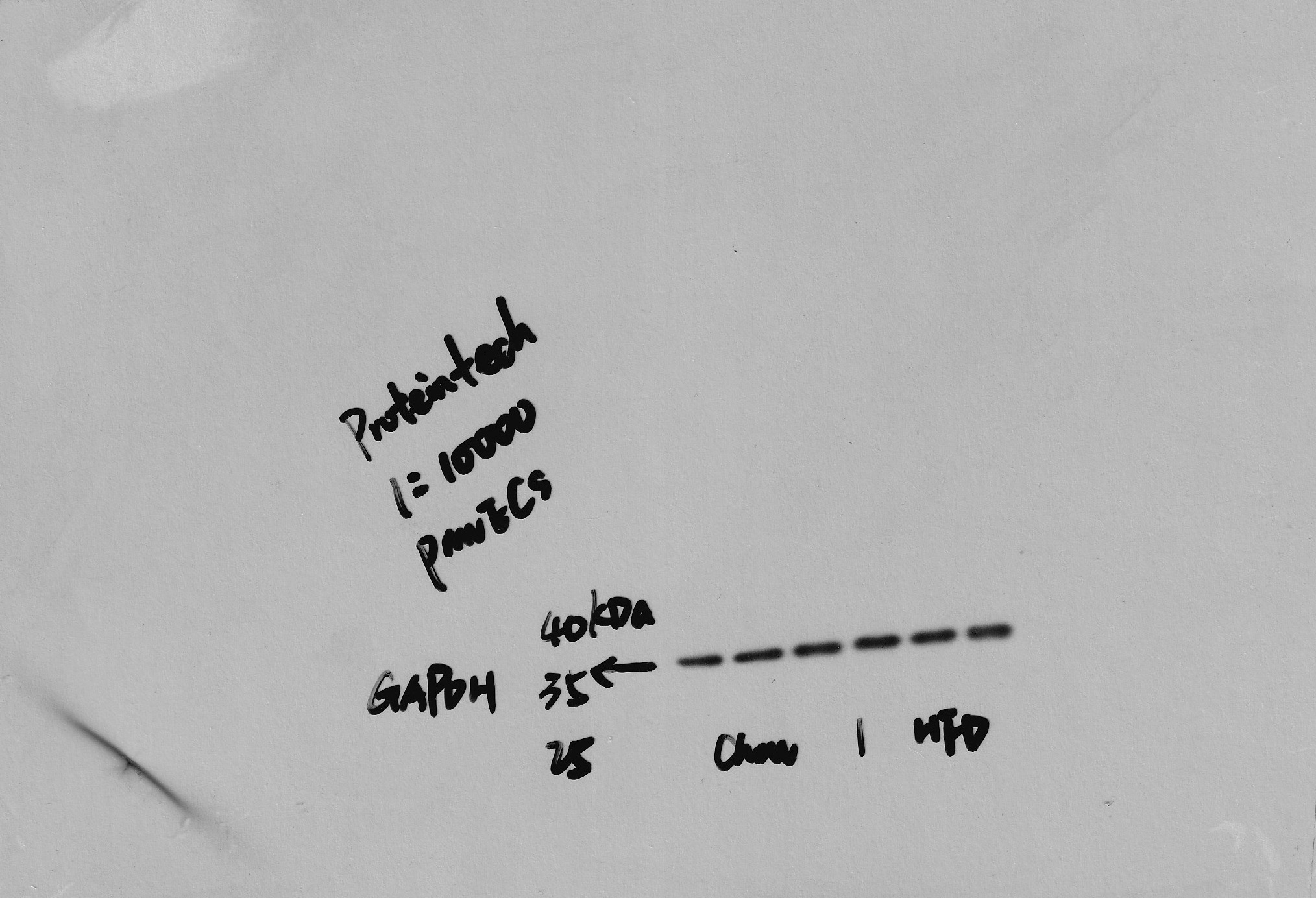

Supplement: Supplementary file 2 — Supplementary Material 2 [file 12931_2024_2827_MOESM2_ESM.jpg]

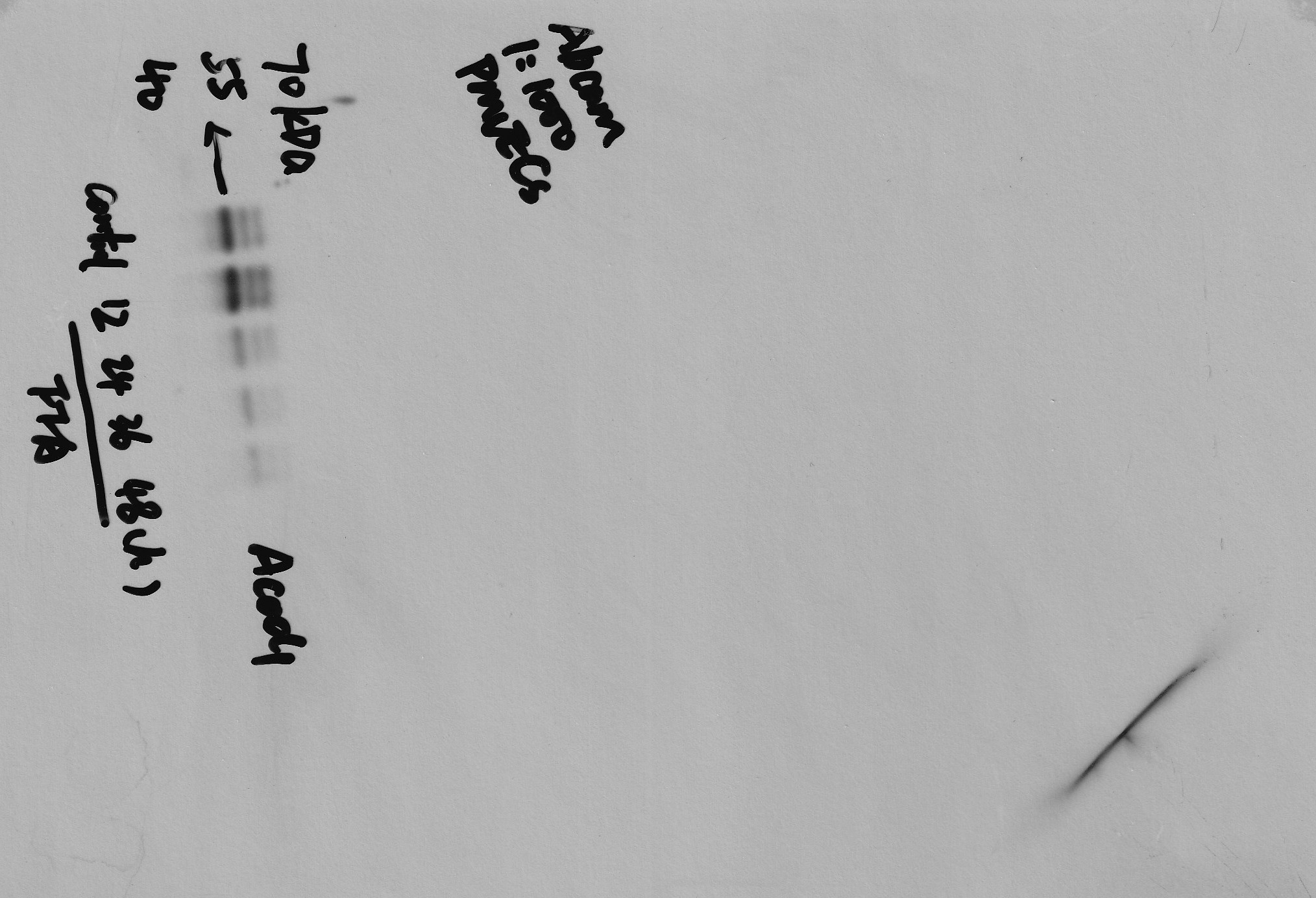

Supplement: Supplementary file 3 — Supplementary Material 3 [file 12931_2024_2827_MOESM3_ESM.jpg]

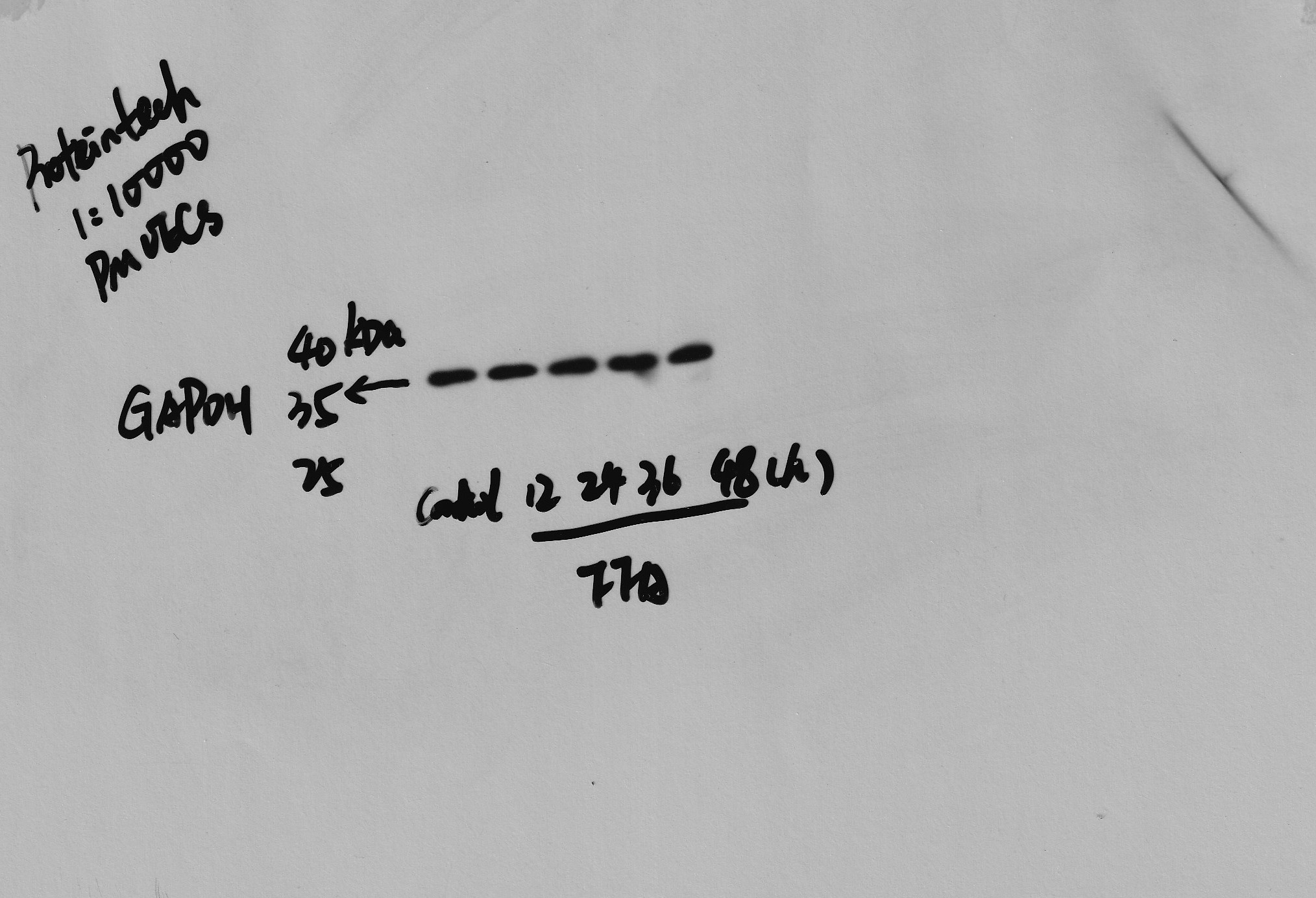

Supplement: Supplementary file 4 — Supplementary Material 4 [file 12931_2024_2827_MOESM4_ESM.jpg]

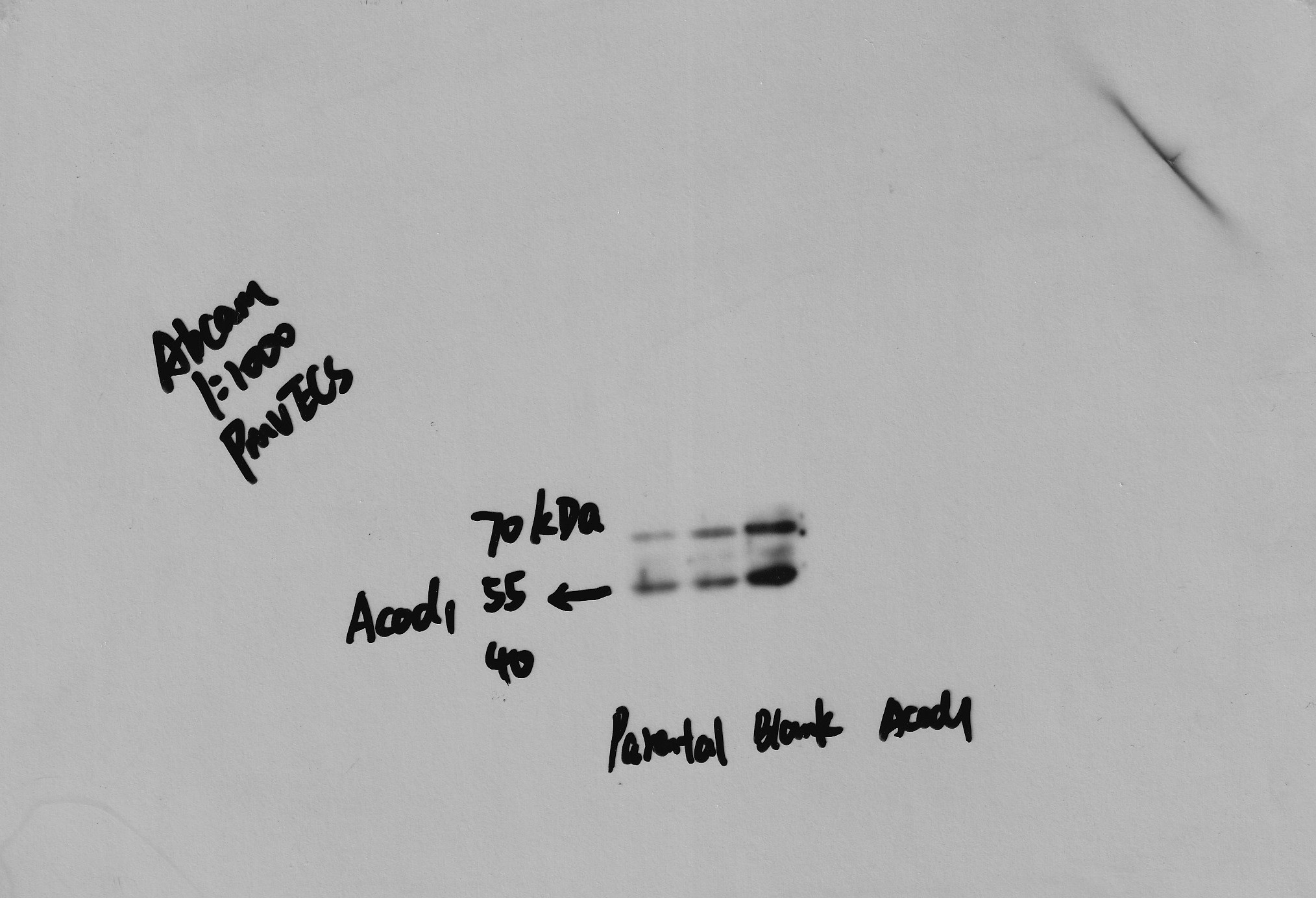

Supplement: Supplementary file 5 — Supplementary Material 5 [file 12931_2024_2827_MOESM5_ESM.jpg]

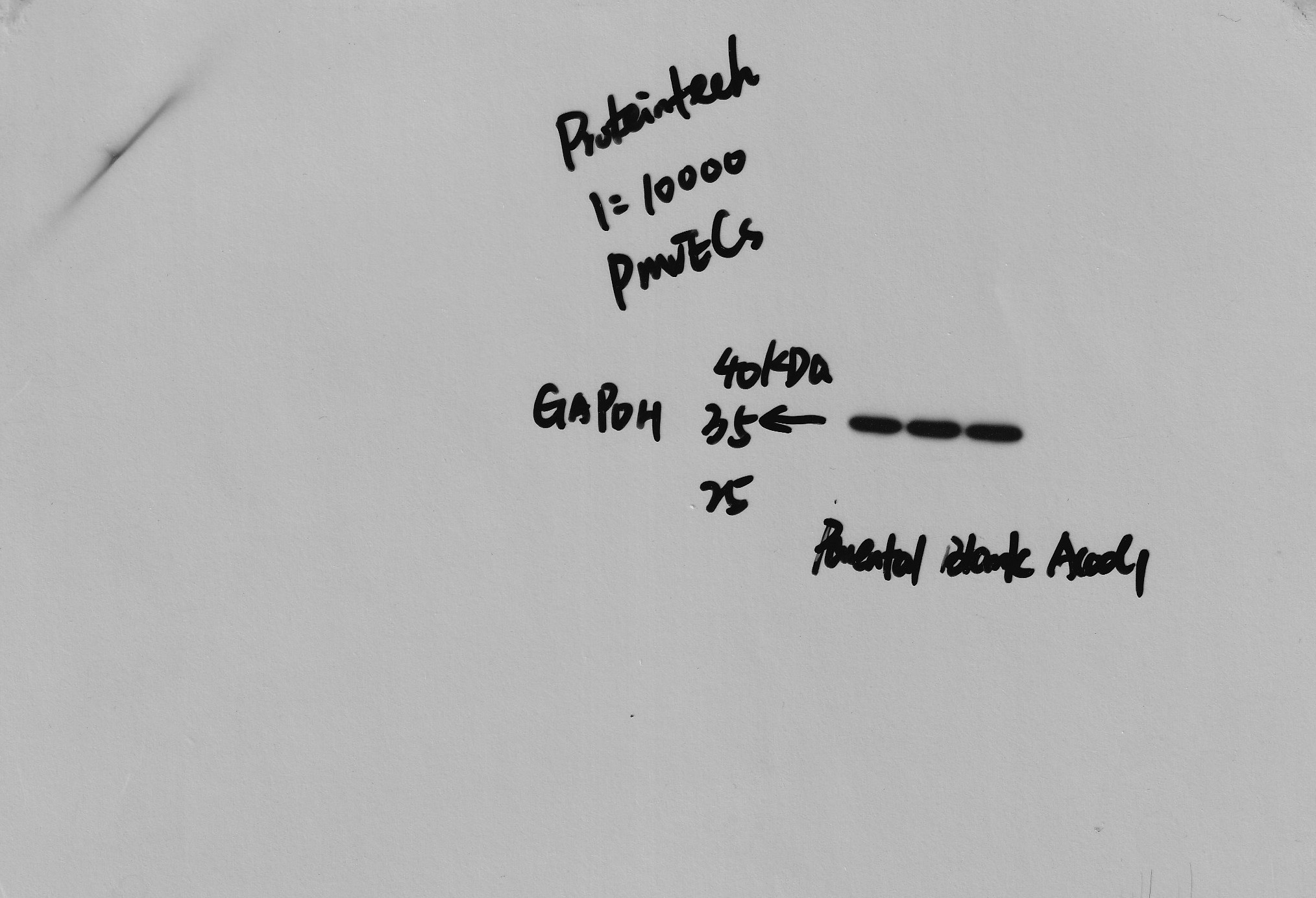

Supplement: Supplementary file 6 — Supplementary Material 6 [file 12931_2024_2827_MOESM6_ESM.jpg]

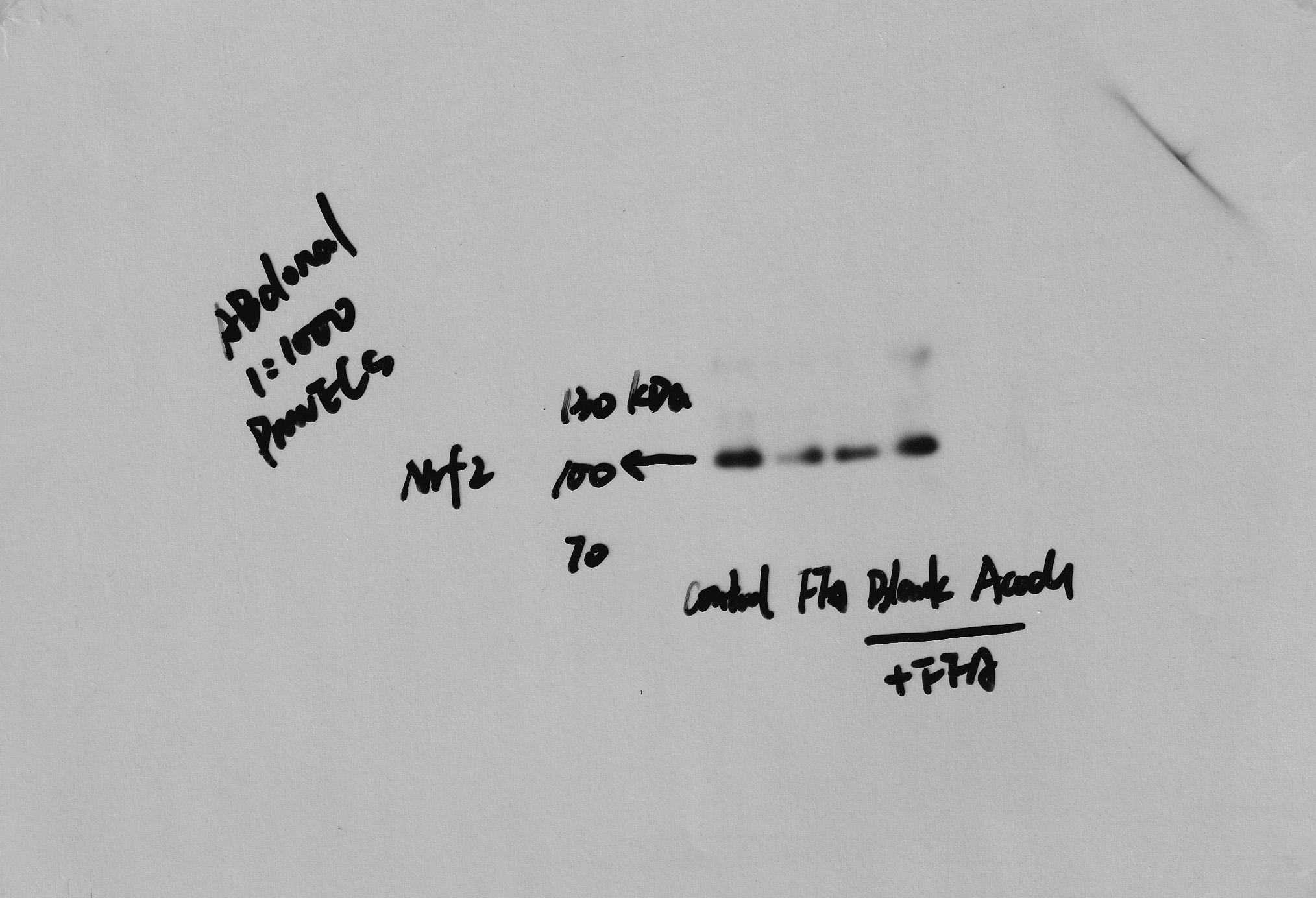

Supplement: Supplementary file 7 — Supplementary Material 7 [file 12931_2024_2827_MOESM7_ESM.jpg]

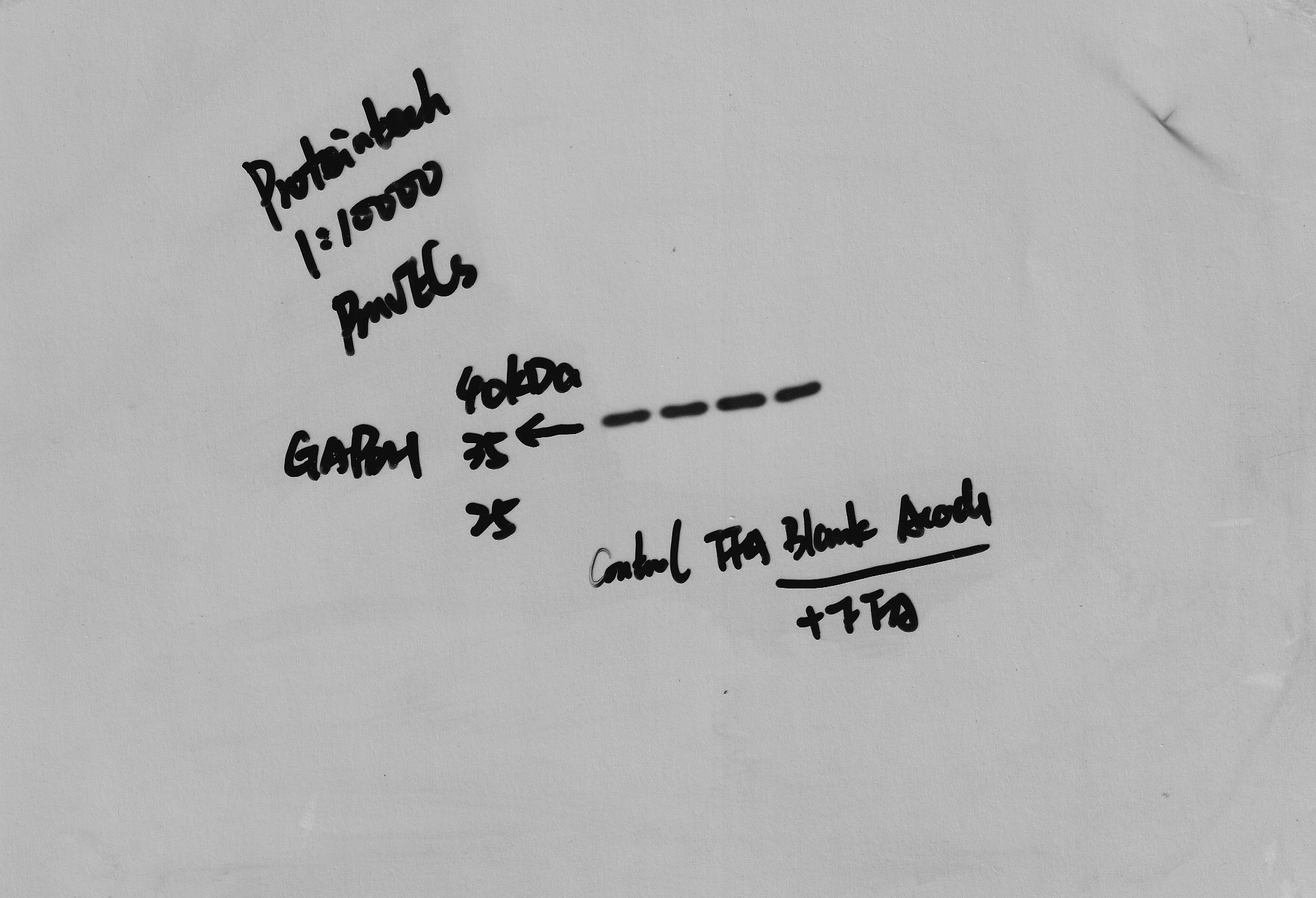

Supplement: Supplementary file 8 — Supplementary Material 8 [file 12931_2024_2827_MOESM8_ESM.jpg]

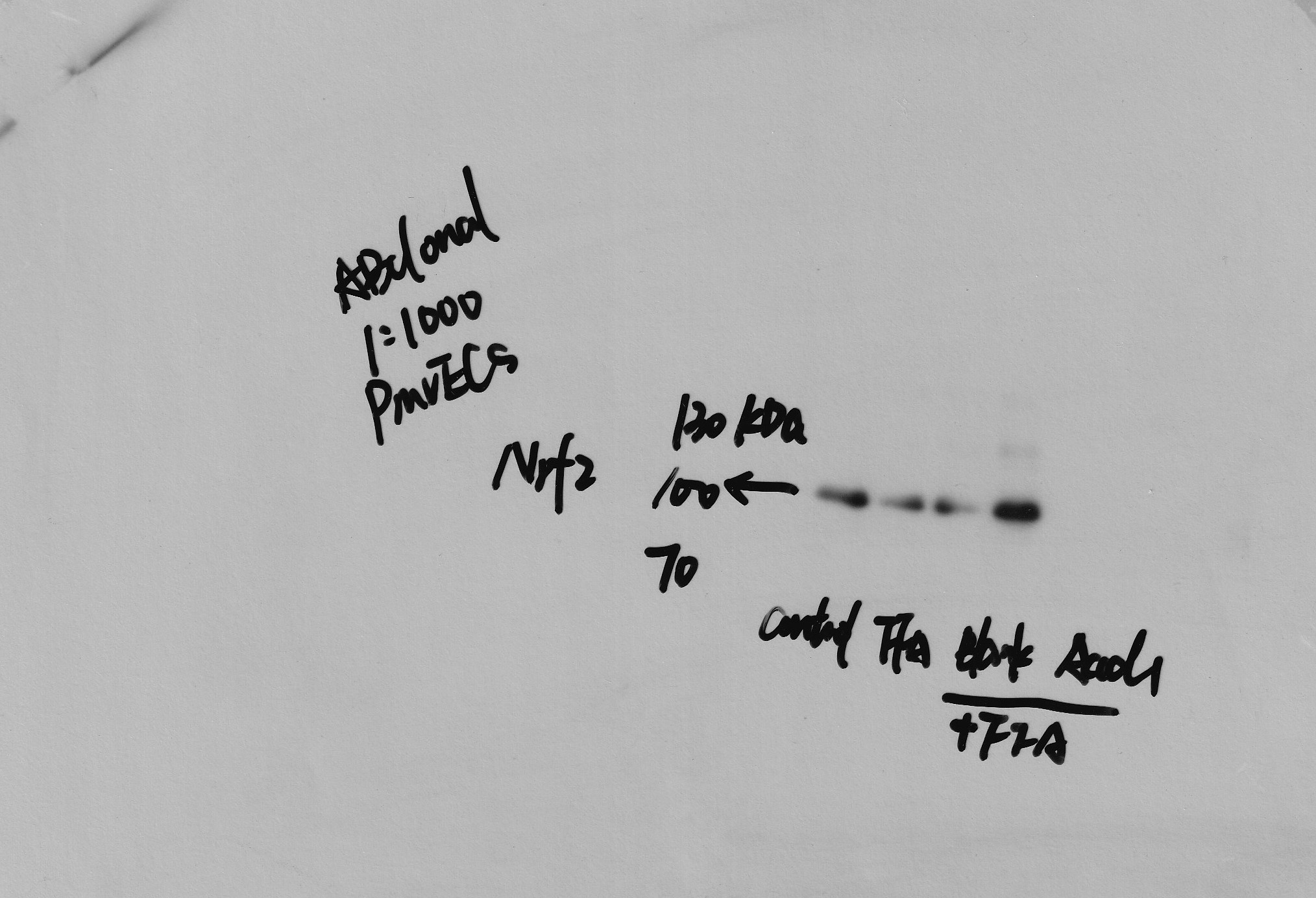

Supplement: Supplementary file 9 — Supplementary Material 9 [file 12931_2024_2827_MOESM9_ESM.jpg]

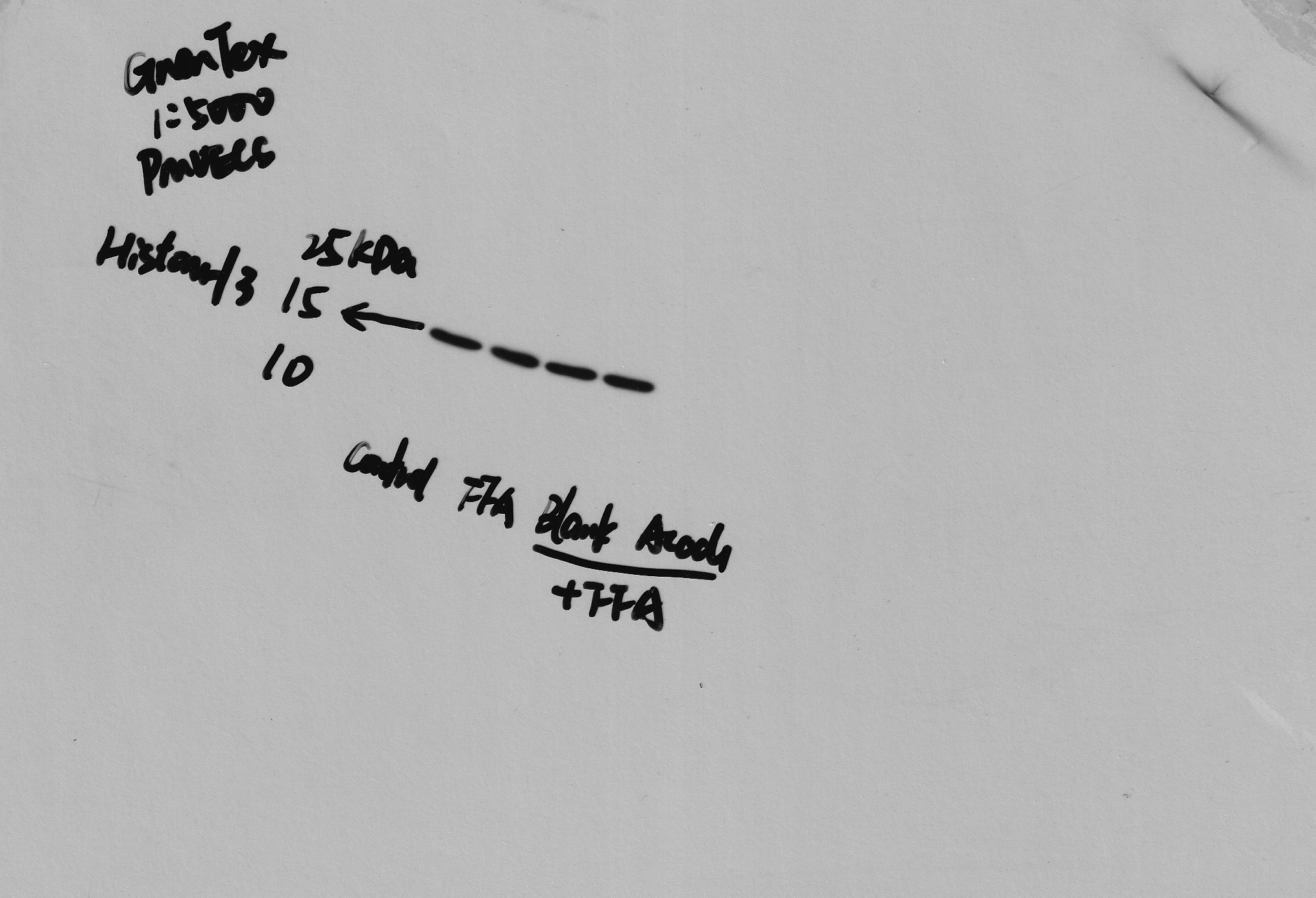

Supplement: Supplementary file 10 — Supplementary Material 10 [file 12931_2024_2827_MOESM10_ESM.jpg]

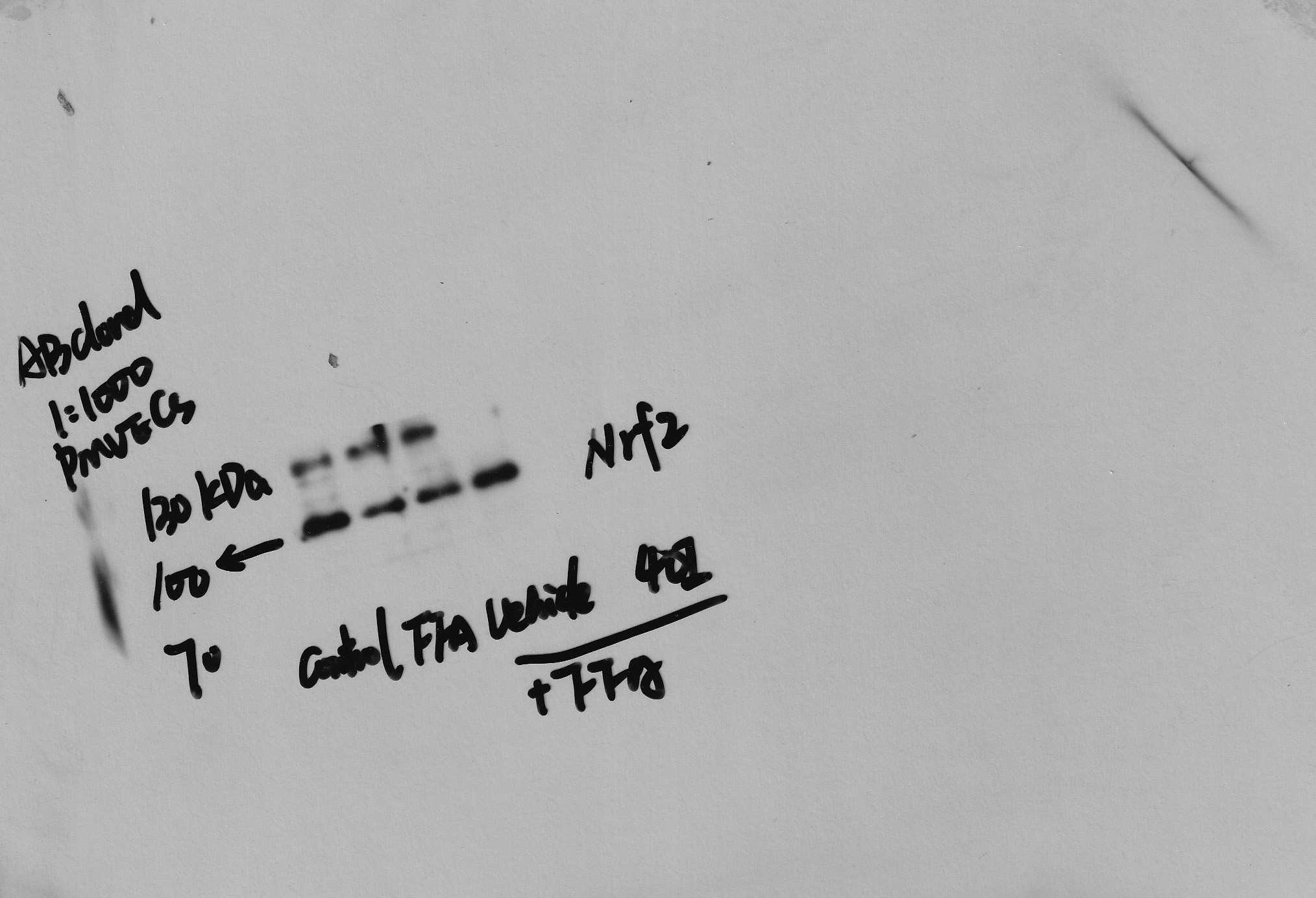

Supplement: Supplementary file 11 — Supplementary Material 11 [file 12931_2024_2827_MOESM11_ESM.jpg]

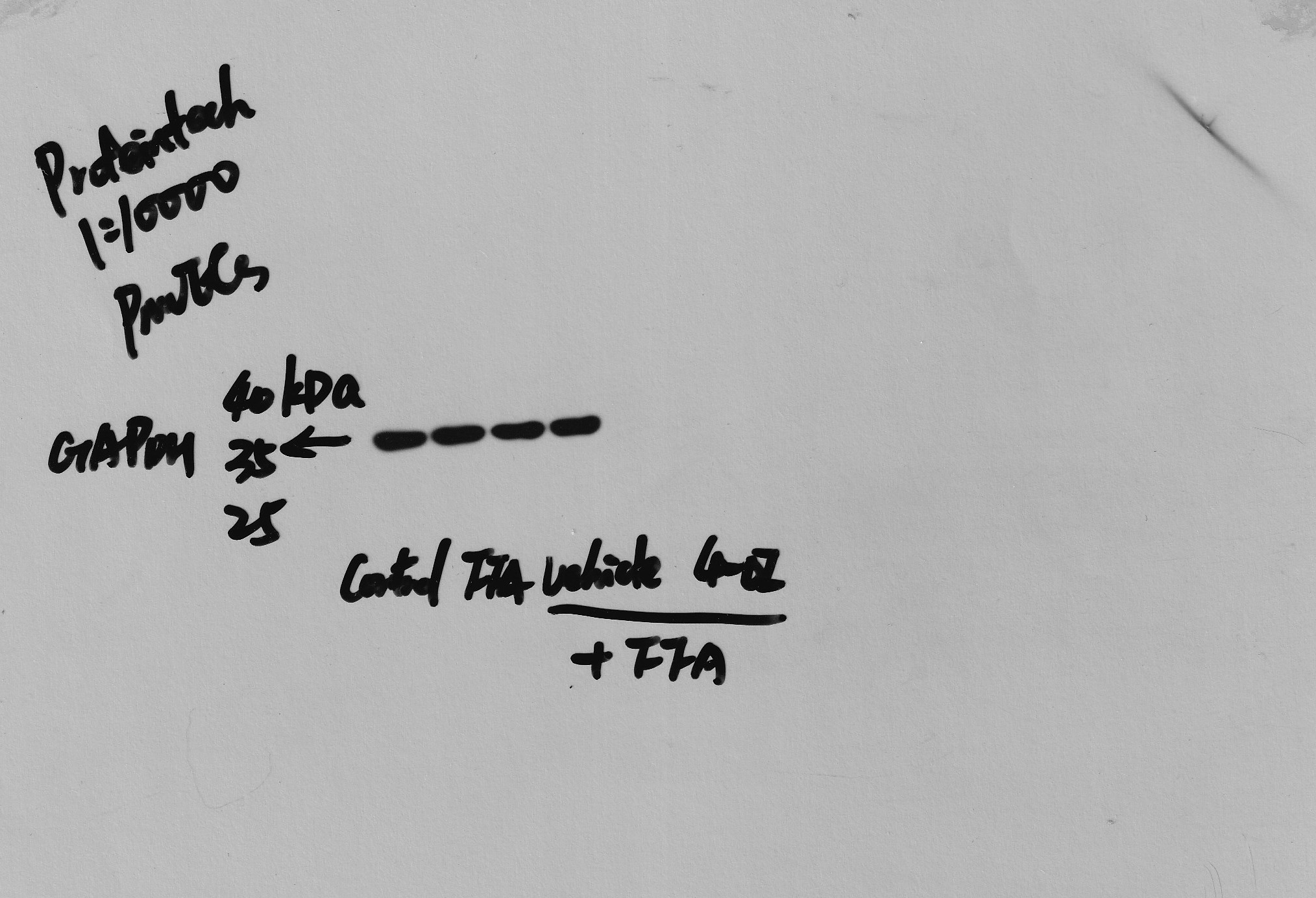

Supplement: Supplementary file 12 — Supplementary Material 12 [file 12931_2024_2827_MOESM12_ESM.jpg]

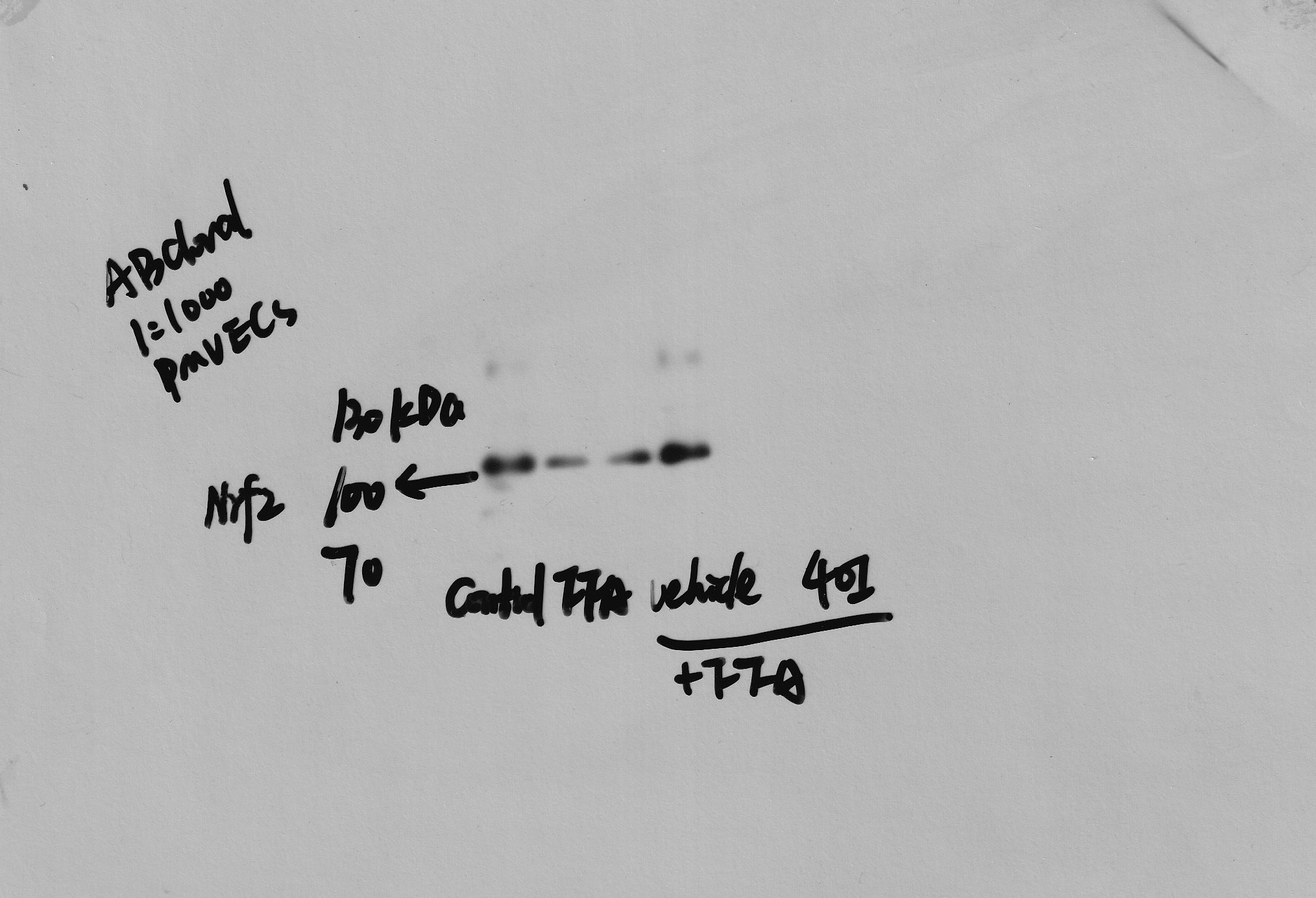

Supplement: Supplementary file 13 — Supplementary Material 13 [file 12931_2024_2827_MOESM13_ESM.jpg]

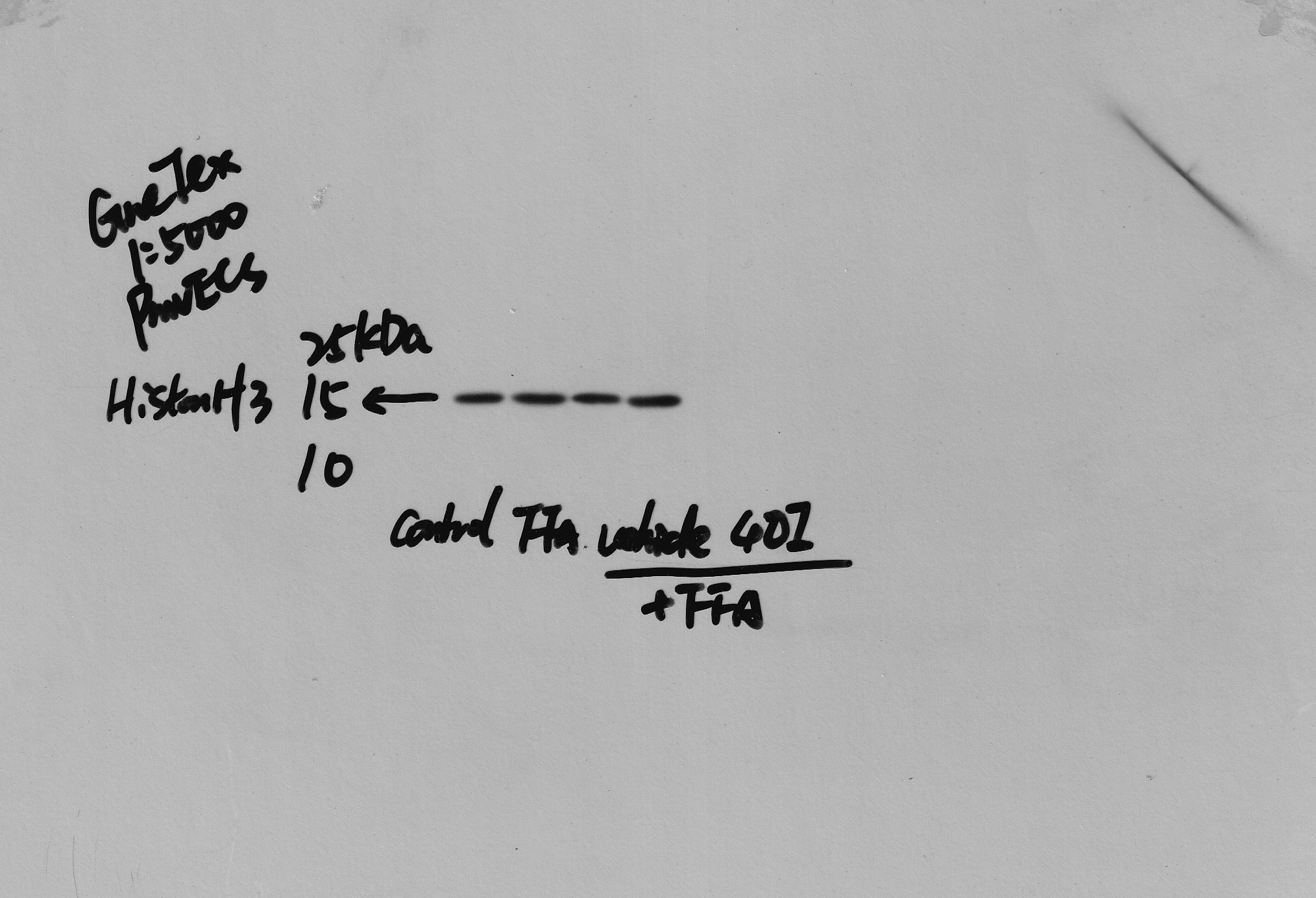

Supplement: Supplementary file 14 — Supplementary Material 14 [file 12931_2024_2827_MOESM14_ESM.jpg]

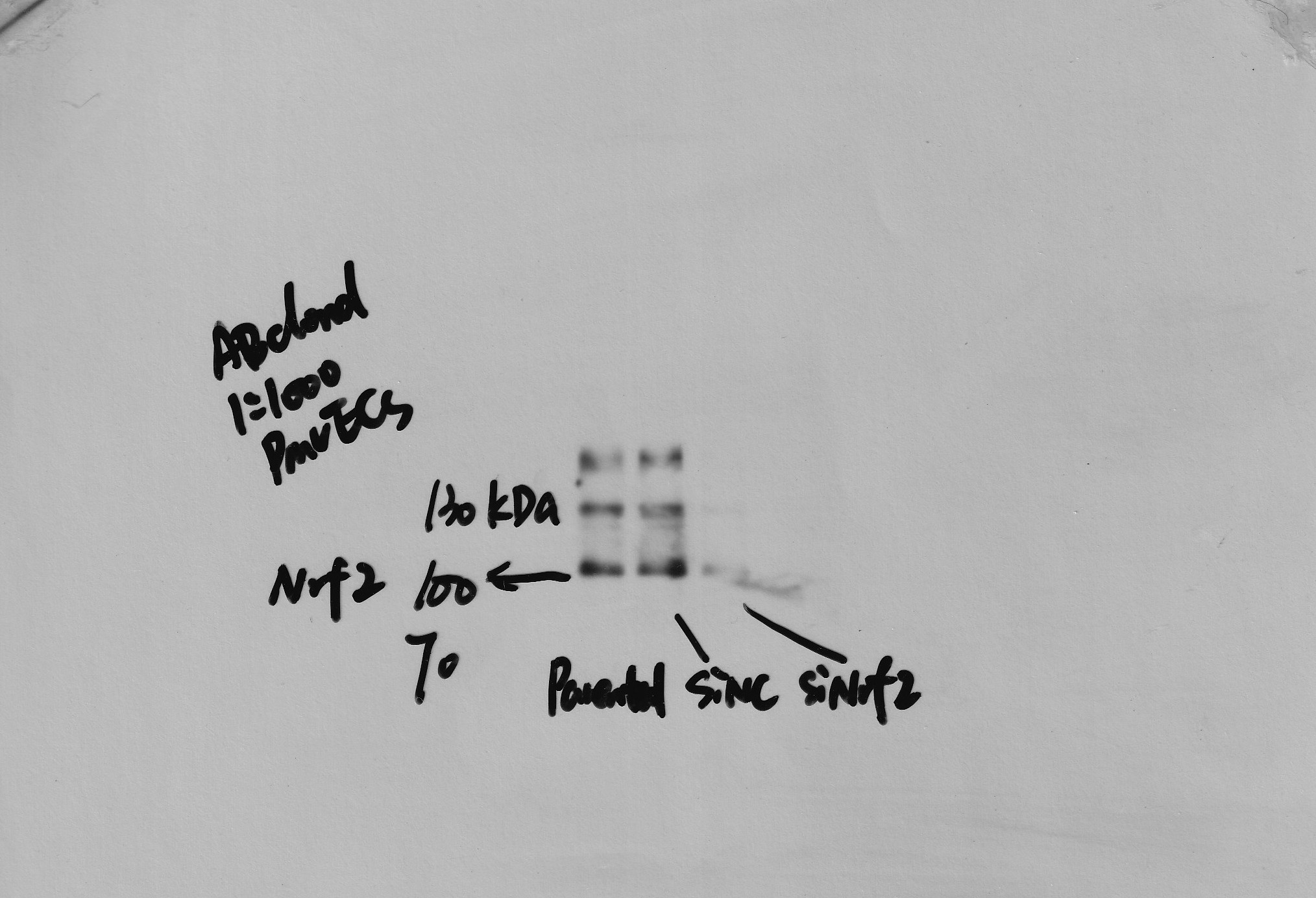

Supplement: Supplementary file 15 — Supplementary Material 15 [file 12931_2024_2827_MOESM15_ESM.jpg]

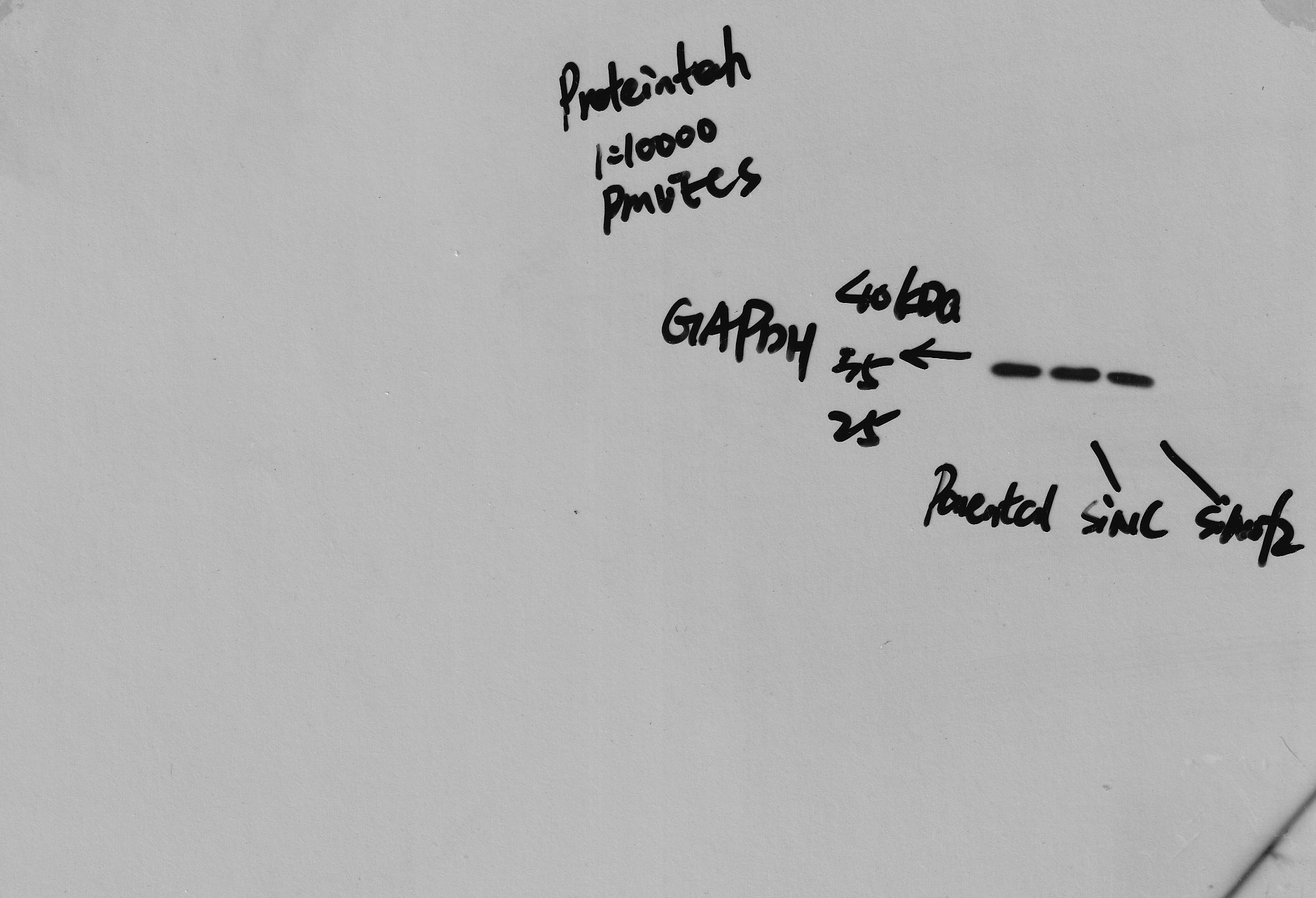

Supplement: Supplementary file 16 — Supplementary Material 16 [file 12931_2024_2827_MOESM16_ESM.jpg]

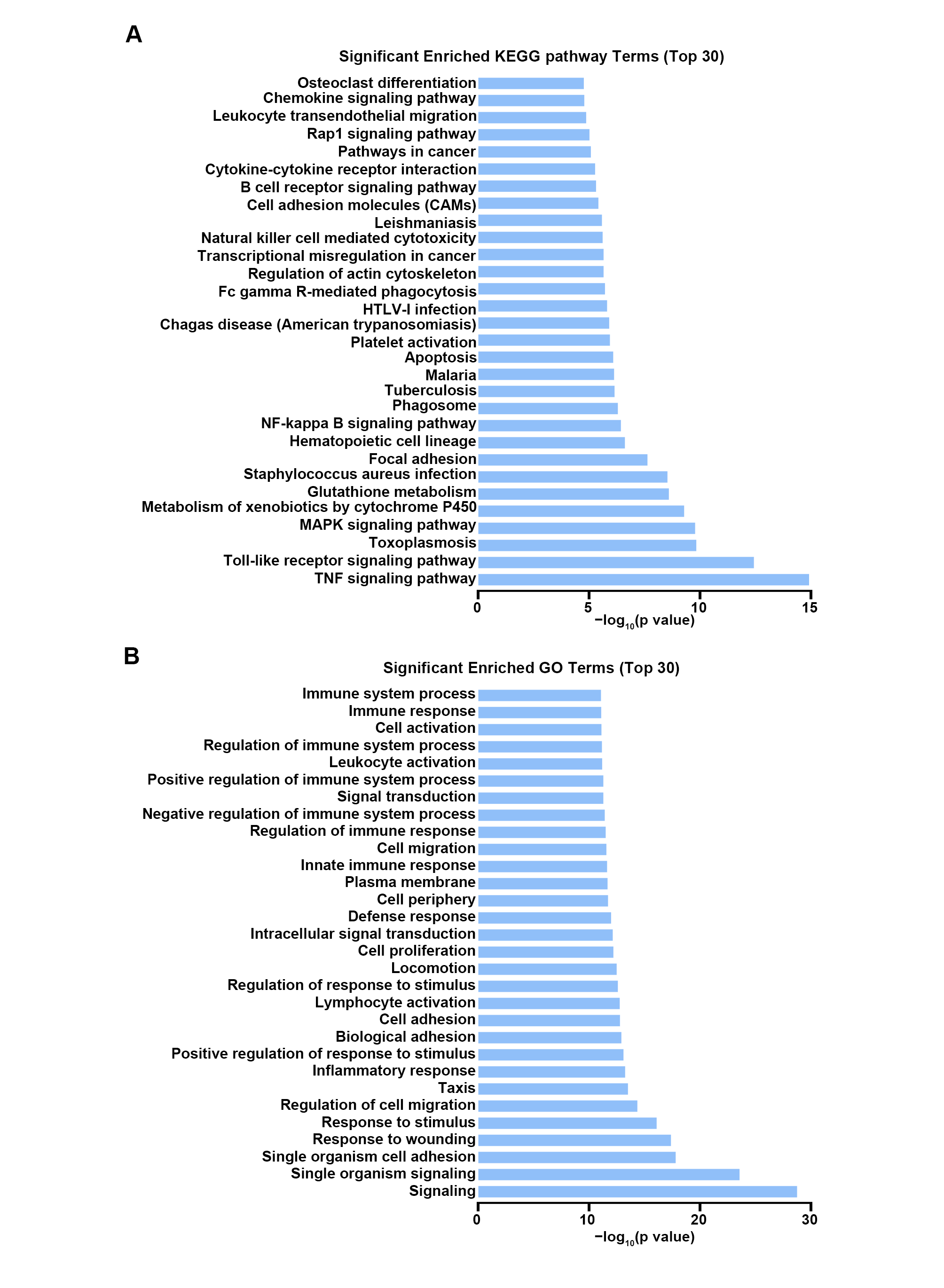

Supplement: Supplementary file 17 — Supplementary Material 17 [file 12931_2024_2827_MOESM17_ESM.png]
